# A Computational Investigation of the Substituent Effects on Geometric, Electronic, and Optical Properties of Siloles and 1,4-Disilacyclohexa-2,5-dienes

**DOI:** 10.3390/molecules22030370

**Published:** 2017-02-28

**Authors:** Aleksandra V. Denisova, Julius Tibbelin, Rikard Emanuelsson, Henrik Ottosson

**Affiliations:** 1 Department of Chemistry–Ångström Laboratory, Uppsala University, Box 523, 75120 Uppsala, Sweden; aleksandra.denisova@kemi.uu.se; 2 Department of Chemistry–BMC, Uppsala University, Box 576, 75123 Uppsala, Sweden; julius.tibbelin@me.com; 3 Nanotechnology and Functional Materials, Department of Engineering Sciences, Uppsala University, Box 534, 75121 Uppsala, Sweden; rikard.emanuelsson@angstrom.uu.se

**Keywords:** 1,4-disilacyclohexa-2,5-diene, cyclobutadisilole, silole, substituent effects, cross-hyperconjugation

## Abstract

Thirty two differently substituted siloles **1a**–**1p** and 1,4-disilacyclohexa-2,5-dienes **2a**–**2p** were investigated by quantum chemical calculations using the PBE0 hybrid density functional theory (DFT) method. The substituents included σ-electron donating and withdrawing, as well as π-electron donating and withdrawing groups, and their effects when placed at the Si atom(s) or at the C atoms were examined. Focus was placed on geometries, frontier orbital energies and the energies of the first allowed electronic excitations. We analyzed the variation in energies between the orbitals which correspond to HOMO and LUMO for the two parent species, here represented as Δε_HL_, motivated by the fact that the first allowed transitions involve excitation between these orbitals. Even though Δε_HL_ and the excitation energies are lower for siloles than for 1,4-disilacyclohexa-2,5-dienes the latter display significantly larger variations with substitution. The Δε_HL_ of the siloles vary within 4.57–5.35 eV (ΔΔε_HL_ = 0.78 eV) while for the 1,4-disilacyclohexa-2,5-dienes the range is 5.49–7.15 eV (ΔΔε_HL_ = 1.66 eV). The excitation energy of the first allowed transitions display a moderate variation for siloles (3.60–4.41 eV) whereas the variation for 1,4-disilacyclohexa-2,5-dienes is nearly doubled (4.69–6.21 eV). Cyclobutadisiloles combine the characteristics of siloles and 1,4-disilacyclohexa-2,5-diene by having even lower excitation energies than siloles yet also extensive variation in excitation energies to substitution of 1,4-disilacyclohexa-2,5-dienes (3.47–4.77 eV, variation of 1.30 eV).

## 1. Introduction

Small cyclic π-conjugated compounds with saturated silicon atoms in their rings are an interesting class of compounds, often investigated for their optoelectronic properties [1,2,3,4,5,6,7,8,9,10,11,12]. Here, siloles, i.e., silacyclopenta-2,4-dienes, have received the greatest attention and their properties can be tuned by variation of the electron donating (EDG) and electron withdrawing (EWG) strengths of the ring substituents [9]. Among their usage in organic electronics can particularly be noted the recent studies on variously substituted siloles for organic light-emitting diodes (OLEDs) [4,5,6,7,8].

**Figure 1:**
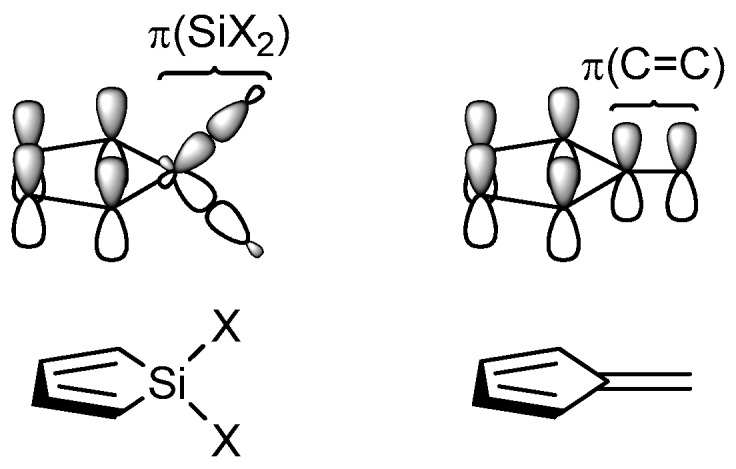
Local π(SiR_2_) fragment orbitals interact in a similar way as exocyclic local π(C=C) orbitals, giving an electronic structure similarity between siloles and pentafulvenes.

Inspired by Mulliken’s seminal studies on hyperconjugation [13,14], we have explored saturated silicon fragments (SiX_2_) inserted between two π-conjugated segments, as found in siloles, and shown that the SiX_2_ fragment contributes with local π(SiR_2_) and π*(SiR_2_) orbitals in a similar way as a geminally connected C=C double bond provides local π(CC) and π*(CC) orbitals [15]. We call this interaction cross-hyperconjugation since it is analogous to regular cross-π-conjugation. Experimental comparisons of electron transfer rates as well as computations of the electron transport through cross-hyperconjugated and cross-π-conjugated segments confirm these similarities [16,17]. Applying the cross-hyperconjugation rational to siloles suggests that siloles can be considered as analogous to the cross-π-conjugated pentafulvenes (Figure 1). Indeed, similar as pentafulvenes, siloles behave as “aromatic chameleons” [18], giving them the ability to adapt their electronic structures to the different π-electron counting rules for aromaticity in the electronic ground state (S_0_) and the first ππ* excited states (T_1_ and S_1_), as given by Hückel’s and Baird’s rules, respectively [19,20,21,22,23]. Utilizing the fact that siloles are cross-hyperconjugated “aromatic chameleons” we could rationalize the substituent effects on the energies of the lowest singlet and triplet excited states [16]. 

During our studies of cross-hyperconjugation another small silacycle, the 1,4-disilacyclohexa-2,5-diene, attracted our interest as a potential complement to siloles in optoelectronic applications [24,25]. Using both experiments and computations we earlier studied its electronic properties, focusing on groups with different tetrel elements as substituents on the two saturated Si ring atoms [26,27]. Although the relative synthetic availability of siloles makes them attractive synthetic targets, the recent account by Stratakis and co-workers shows that 1,4-disilacyclohexa-2,5-dienes are also synthetically obtainable [28]. Thus, we now probed if the 1,4-disilacyclohexa-2,5-dienes respond to substitution similar as siloles, i.e., do electron withdrawing and electron donating groups at the Si or C atoms have the same effects?

In siloles the influence is generally largest for substituents at the 2- and 5-positions since substituents at these positions can exhibit strong π-interaction with the silole core; strong π-donors destabilize HOMO while strong π-acceptors stabilize LUMO [9]. On the other hand, it is known that substituents at Si only have minor effects on optical properties (absorption and fluorescence characteristics) [9]. Due to synthetic limitations, substitution at 3- and 4-positions is not equally thoroughly examined as substitution at the other positions. However, DFT calculations of 2,3,4,5-tetraarylsiloles reveal that substituents at those positions give considerably smaller extensions of both HOMO and LUMO when compared to similar substitution at the 2- and 5-positions [2,29,30,31,32,33,34]. 

Using quantum chemically calculated properties, such as electronic transitions and geometric parameters, herein we analyze how the substituent patterns in the different compounds are linked to their cross-hyperconjugation. Are the substituent effects more pronounced in the siloles or in the 1,4-disilacyclohexa-2,5-dienes? And how could one go about to design compounds that combine the beneficial features of siloles with those of 1,4-disilacyclohexa-2,5-dienes? 

## 2. Results and Discussion

**Figure 2:**
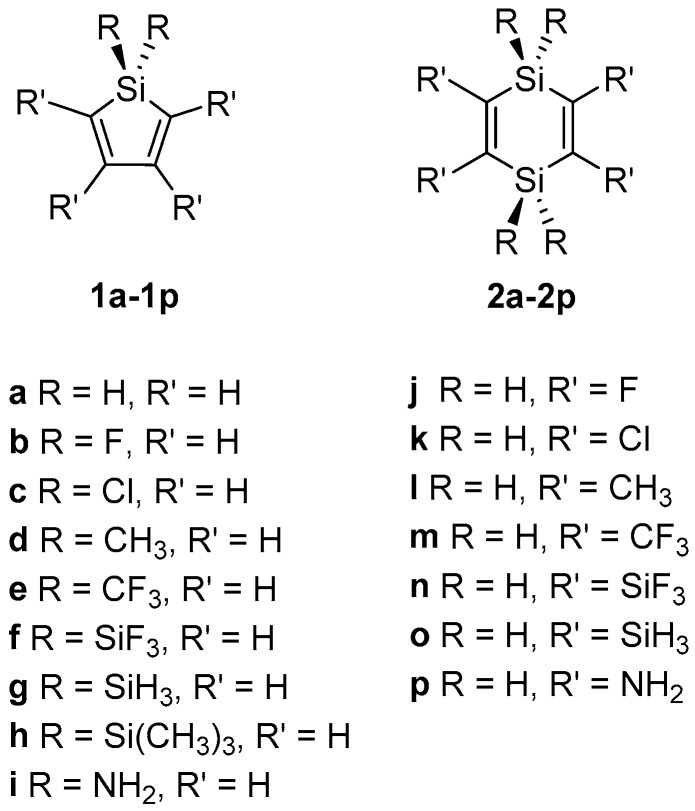
The compounds contained in the present study.

The comparison of the variously substituted **1** and **2** (Figure 2) is based on both geometric and electronic structural data, and we discuss each of the properties (geometrical data, molecular orbital data, and electronic excitation energies) separately. Rather than comparing a silole having a particular substituent against the 1,4-disilacyclohexa-2,5-diene with the same substituents we analyze the spread in the values of the selected properties for the chosen set of substituents. We then primarily discuss the compounds at the end points in the spreads. For tabulations of the properties of each individual silole and 1,4-disilacyclohexa-2,5-diene, see the Supporting Information.

### 2.1. Geometric Structure

**Figure 3:**
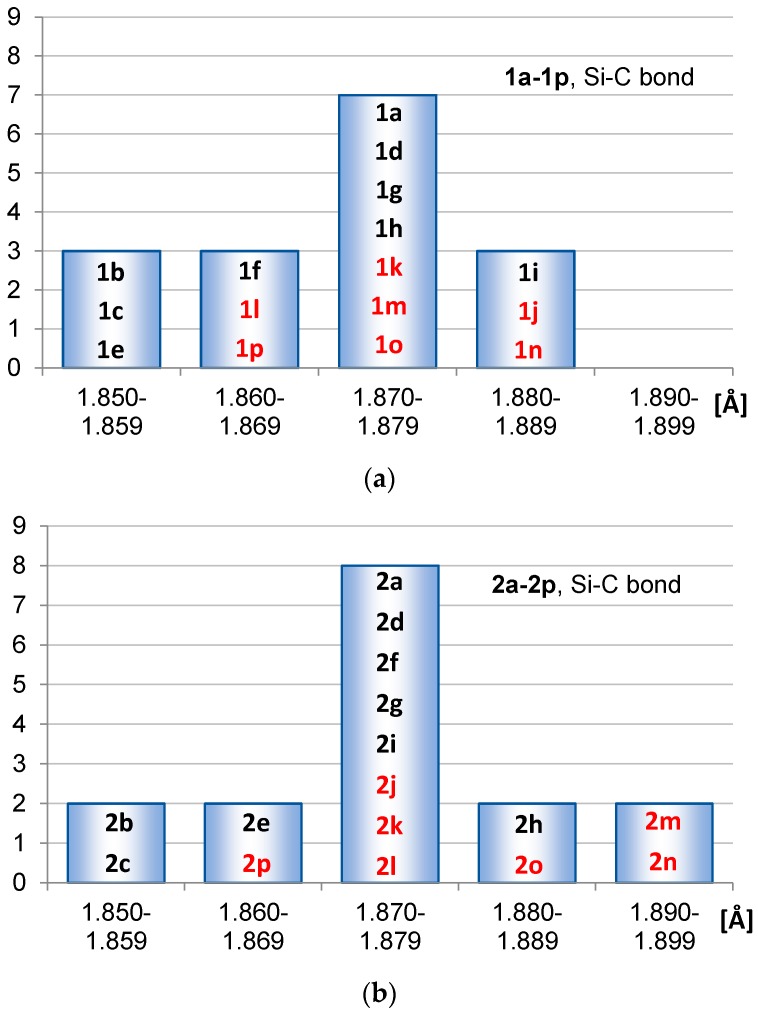
Distributions of SiC(ring) bond lengths of (**a**) **1a**–**1p** and (**b**) **2a**–**2p** calculated at PBE0/6-31G(d) level. The compound numbers substituted at the Si atoms are displayed in black and those substituted at the C atoms in red.

**Figure 4:**
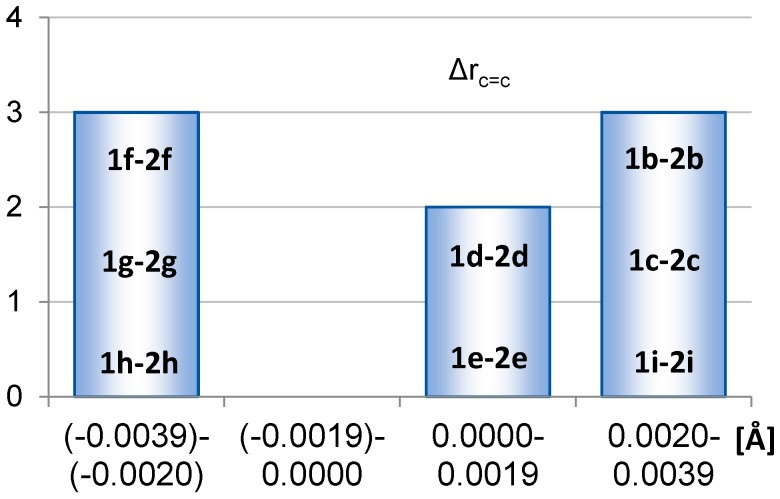
Distribution of Δr_c=c_ = [r_c=c_(**1a**) − r_c=c_(**2a**)] − [r_c=c_(**1x**) − r_c=c_(**2x**)] values calculated at PBE0/6-31G(d) level, with **x** = **b** to **i**.

**Figure 5:**
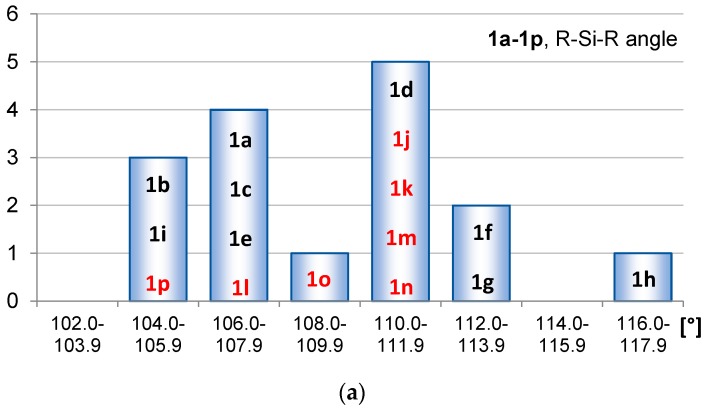

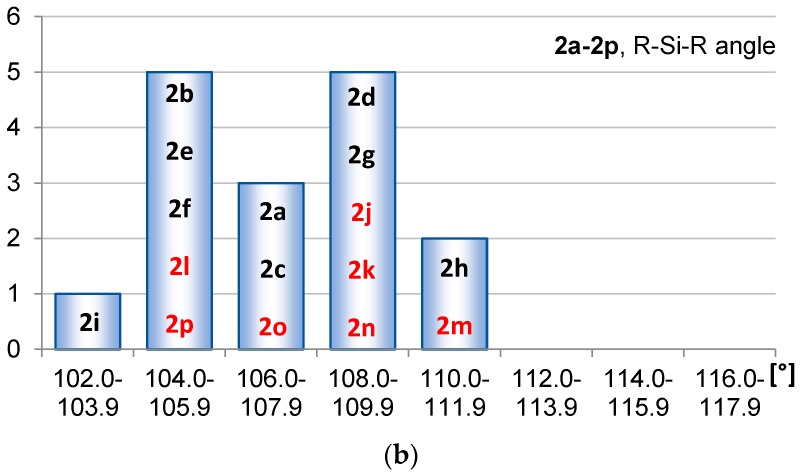
Distributions of the R-Si-R angles of (**a**) **1a**–**1p** and (**b**) **2a**–**2p** given in degrees and calculated at the PBE0/6-31G(d) level. The compound numbers substituted at the Si atoms are displayed in black and those substituted at the C atoms in red.

As indicators of hyperconjugation we examined SiC(ring) and C=C bond lengths as well as R-Si-R angles. The spread in these geometrical parameters are displayed in the bar diagrams of Figure 3, Figure 4 and Figure 5. Improved conjugation generally leads to elongation of double bonds and shortening of single bonds, and hyperconjugation also has this effect [23]. In the analysis herein we separated between the siloles and 1,4-disilacyclohexa-2,5-dienes having substituents at Si (**1b**–**1i** and **2b**–**2i**) and those having substituents at C (**1j**–**1p** and **2j**–**2p**). The latter compounds can in addition to the electronic effects also display geometric distortions that are caused by steric congestion between substituents. For that reason we do not analyze the C=C bond lengths of these compounds, except in a few selected cases.

The average SiC(ring) bond lengths in **1a**–**1p** and **2a**–**2p** are 1.871 and 1.874 Å, respectively, and the shorter average bond length in the siloles than in the 1,4-disilacyclohexa-2,5-dienes is reflected in the spread within the bond length intervals (Figure 3). Both among the siloles and the 1,4-disilacyclohexa-2,5-dienes are species with SiC(ring) bond lengths in the range 1.850–1.859 Å. However, no silole has SiC bonds in the interval 1.890–1.899 Å whereas two 1,4-disilacyclohexa-2,5-dienes have (**2m** and **2n**). The generally shorter SiC bonds in **1a**–**1p** than in **2a**–**2p** suggest stronger hyperconjugation between the single SiR_2_ segment and the diene segment in siloles than between the SiR_2_ segments and the two formally non-conjugated C=C bonds in the 1,4-disilacyclohexa-2,5-dienes.

When considering the effect of the substituents at Si (the R groups) it is clear that σ-electron withdrawing groups lead to the shortest SiC(ring) bonds both in siloles and in 1,4-disilacyclohexa-2,5-dienes. On the other hand, R = NH_2_ in siloles (compound **1i**) and R = SiMe_3_ in 1,4-disilacyclohexa-2,5-dienes (compound **2h**) lead to the longest ones. With regard to the effect of the R′ substituent on the SiC(ring) bond length we only considered the effect for 1,4-disilacyclohexa-2,5-dienes as the steric congestion in a few of the siloles is extensive. Among the substituents at the C=C bonds of the 1,4-disilacyclohexa-2,5-diene the trifluoromethyl groups, leading to **2m**, give SiC(ring) bonds which are elongated when compared to **2a**. With R′ = NH_2_ (compound **2p**) they are the shortest. From this one can deduce that among the 1,4-disilacyclohexa-2,5-dienes the shortest SiC(ring) bond should be found with R = F and R′ = NH_2_ while the longest would be found with R = SiMe_3_ and R′ = CF_3_. Indeed, this is supported by computations because the 1,4-disilacyclohexa-2,5-diene with R = F and R′ = NH_2_ has a SiC(ring) bond length of 1.840 Å whereas when R = SiMe_3_ and R′ = CF_3_ it is 1.915 Å. 

The formal C=C double bonds of **1a** and **2a** are 1.348 and 1.346 Å, respectively. However, in siloles they are part of the diene segment whereas in 1,4-disilacyclohexa-2,5-dienes they are instead two isolated double bonds. For that reason we do not compare the C=C bonds directly but instead compare the deviations in bond lengths against those of the respective parent compounds. Thus, we consider Δr_c=c_ = [r_c=c_(**1a**) − r_c=c_(**2a**)] − [r_c=c_(**1x**) − r_c=c_(**2x**)] (Figure 4) where positive values show that a C=C double bond lengthening is more pronounced in 1,4-disilacyclohexa-2,5-dienes, while negative values correspond to situations with more elongated double bonds in siloles. The species investigated only include those with substituents at Si so as to exclude effects caused by steric congestion. Here it should be noted that siloles with small electron withdrawing substituents at the double bonds can show shortening of these bonds. E.g., the fluoro substituents in **1j** lead to the shortest C=C double bonds among all siloles considered here (1.341 Å), and the chloro and trifluoromethyl substituents (**1k** and **1m**) reveal double bonds of 1.349 Å, which are only slightly longer than those of **1a**. 

For compounds with electron donating silyl groups as substituents at the silicon (**1f**–**1h**), the elongations of the C=C bonds are more pronounced in the siloles than in the 1,4-disilacyclohexa-2,5-dienes. Conversely, σ-electron withdrawing groups as R lead to more significant shortenings of the double bonds in siloles than in the 1,4-disilacyclohexa-2,5-dienes or display similar r_C=C_ values (**1b**–**1e**). Attachment of two amino groups at silicon results in significantly more pronounced double bond shortenings in the silole **1i** than in the 1,4-disilacyclohexa-2,5-diene **2i**.

For the various siloles the R-Si-R angles are found mainly in the range 105.2°–113.8°, but one silole (**1h**, R = SiMe_3_) is found in the interval 116.0°–117.9° (Figure 5a). The average value is 109.4°. Interestingly, the R-Si-R angles of the siloles cluster into two groups. To some extent the R-Si-R angles are also affected by steric bulk of the R groups (particularly applicable to **1h**). Yet, for the siloles with R = H (**1j**–**1p**) there is still a spread in the angles of 5.8°, indicating that the variation to a substantial extent is of electronic origin.

For the full set of 1,4-disilacyclohexa-2,5-dienes, the R-Si-R angles are found in the smaller range 103.7°–110.9°, with an average value of 107.2°. The larger average R-Si-R angle of the siloles could be explained by a general tendency that a smaller bond angle at an atom with tetrahedral bond arrangement obtained through inscription of this atom in a ring is often compensated by a larger bond angle between the two bonds that are not inscribed into the cycle [35,36]. The C(ring)-Si-C(ring) angles of **1a**–**1p** and **2a**–**2p** are found in the range 90.6°–95.0°, i.e., they are significantly smaller than the ideal tetrahedral value, and this results in the large R-Si-R angles observed. Similar as for the siloles, the species **2i**–**2p** have a spread in the H-Si-H angles (Figure 5b), also now revealing the electronic effect of the substituents at the C atoms on this angle. 

For both siloles and 1,4-disilacyclohexa-2,5-dienes it is clear from the bar diagrams that the compounds with σ-withdrawing substituents at Si in general have small R-Si-R angles, although among **2a**–**2p** the species with the smallest R-Si-R angles are those with π-donating substituents (**2i** and **2p**).

### 2.2. Molecular Orbitals

**Figure 6:**
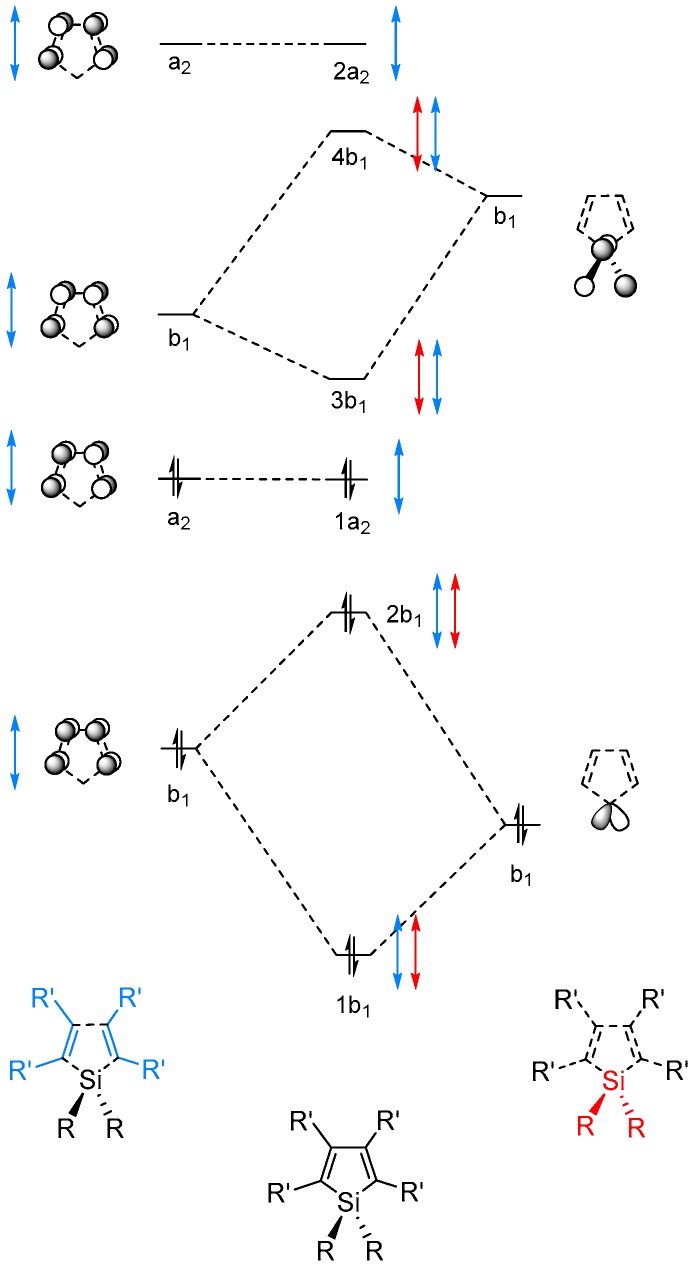
Qualitative molecular orbital (MO) diagram of silole with the lowest few occupied and unoccupied MOs of π-character constructed from suitable fragment orbitals. Red arrows indicate changes in fragment orbital energies in dependence of substituent R and blue arrows indicate changes of substituent R′. The orbitals are labeled in accordance with the irreducible representations of the *C*_2v_ point group.

**Figure 7:**
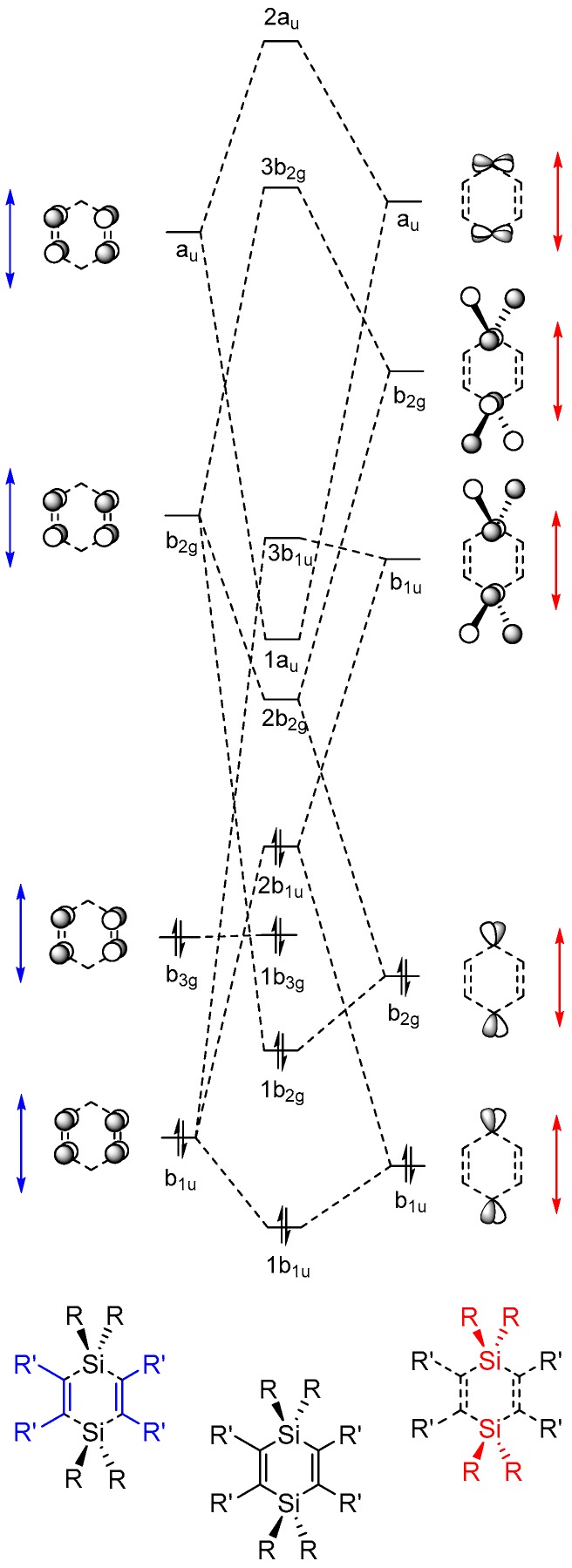
Qualitative molecular orbital (MO) diagrams of 1,4-disilacyclohexa-2,5-diene with the lowest few occupied and unoccupied MOs of π-character constructed from suitable fragment orbitals. Red arrows indicate changes in fragment orbital energies in dependence of substituent R and blue arrows indicate changes of substituent R′. The orbitals are labeled in accordance with the irreducible representations of the *D*_2h_ point group, and the ordering is that of **2a** according to PBE0/6-31G(d) calculations.

The MOs of π-symmetry are constructed qualitatively by regarding the proper symmetry combinations of the π-orbitals of the unsaturated carbon segments, with the bonding and antibonding orbitals of π-character at the single SiR_2_ fragment in siloles (Figure 6) or the two SiR_2_ fragments in 1,4-disilacyclohexa-2,5-dienes (Figure 7). To examine the effect of the substituents on the frontier orbital energies we regard the Kohn-Sham orbitals from the PBE0/6-31G(d) calculations. 

**Figure 8:**
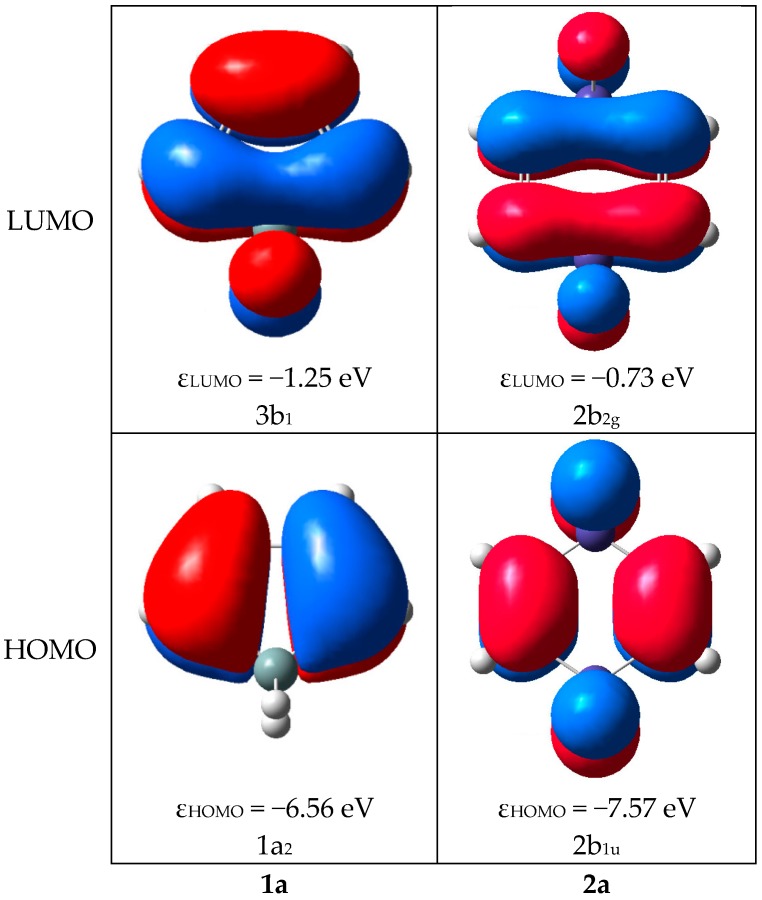
Plots of HOMO and LUMO of **1a** and **2a**, respectively, their orbital energies at PBE0/6-31G(d) level and their symmetry notations and orders.

**Figure 9:**
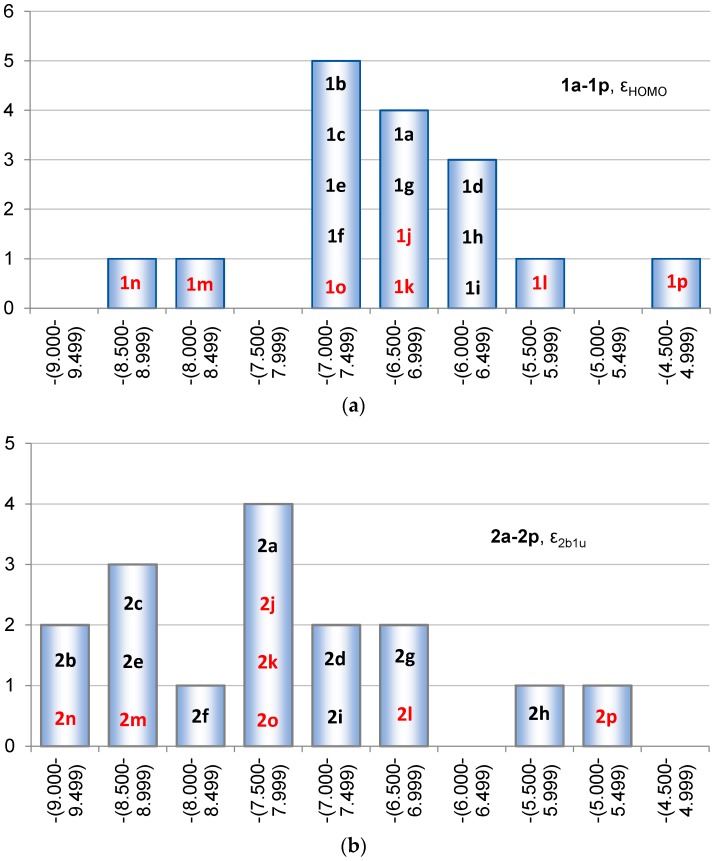
Distribution of (**a**) the ε_HOMO_ for **1a**–**1p** and (**b**) the ε_2b1u_ for **2a**–**2p** (ε_HOMO_ for **2a**). Compound numbers of compounds substituted at the Si atoms in black and those substituted at the C atoms in red.

The HOMO for each of the siloles **1a**–**1p** is of the same type, and it belongs to the either the a or the a_2_ irreducible representation, depending on whether the silole is *C*_2_ or *C*_2v_ symmetric. In **1a** HOMO is the 1a_2_ orbital (Figure 8). The ε_HOMO_ in the siloles is on average −6.78 eV, whereas the span ranges from −8.75 to −4.58 eV with the two extreme values found for **1n** and **1p** (Figure 9), respectively. However, there are only two siloles which have ε_HOMO_ below −8.0 eV (**1m** and **1n**), the others have ε_HOMO_ at energies −7.33 eV or above. Since HOMO lacks contributions from the Si atom, the variation in ε_HOMO_ among **1a**–**1i**, in which R at Si is varied, is merely 1.19 eV (from −7.33 to −6.14 eV), compared to 4.17 eV for the complete series **1a**–**1p**. Thus, the variation in ε_HOMO_ for the siloles is best achieved through substitution at the diene segment, in line with the conclusions by Marder and co-workers who pointed out that 2,5-disubstitution has more pronounced effects on the electronic and optical properties than 1,1-disubstitution [9]. 

It is also noteworthy that the siloles in which the SiC(ring) bonds are the shortest (**1b**, **1c** or **1e**) or the longest (**1i**, **1j** and **1n**) are not among the siloles with particularly low or high ε_HOMO_ values. The silole with the highest-energy HOMO (**1p**) has a small H-Si-H angle, yet, there is no obvious connection between the ε_HOMO_ and the geometrical parameters for the siloles. In contrast, the HOMO-1 orbital (2b_1_) is clearly the orbital with contributions from both the diene and SiR_2_ segments, and which therefore impacts on the interaction. 

**Figure 10:**
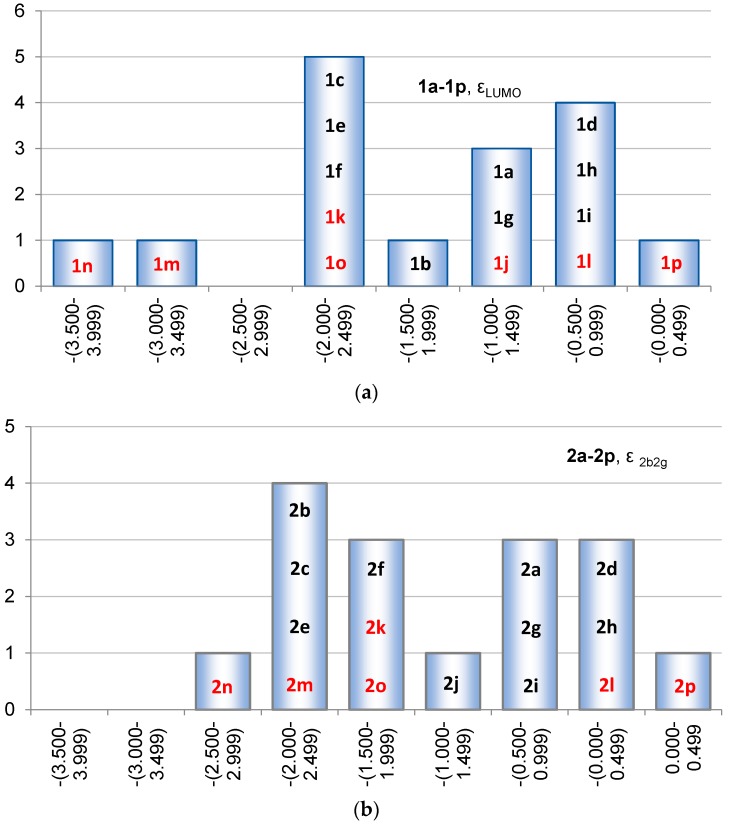
Distribution of (**a**) the ε_LUMO_ for **1a**–**1p** and (**b**) the ε_2b2g_ for **2a**–**2p** (ε_LUMO_ for **2a**). Compound numbers of compounds substituted at the Si atoms in black and those substituted at the C atoms in red.

Furthermore, the LUMOs throughout **1a**–**1p** belong either to the b or the b_1_ irreducible representation, depending on molecular symmetry (*C*_2_ or *C*_2v_). In **1a** LUMO is the 3b_1_ orbital (Figure 8). The average value of ε_LUMO_ among all siloles is −1.70 eV, with the lowest LUMO found for **1n** (ε_LUMO_ = −3.78 eV) followed by **1m** (ε_LUMO_ = −3.25 eV), both having electron withdrawing substituents (trifluorosilyl and trifluromethyl groups) at the diene segment (Figure 10). The highest ε_LUMO_ is found for **1p** (−0.01 eV) having tetraamino substitution at the diene segment. As LUMO has contributions from both the diene and SiR_2_ segments it varies in energy slightly more with substitution at Si (Δε_LUMO_ = 1.32 eV ranging from −2.11 to −0.79 eV) than reported above for the variation in energy of HOMO. However, to achieve a large variation in the ε_LUMO_ of siloles the positions at both the C and Si atoms need to be utilized.

**Figure 11:**
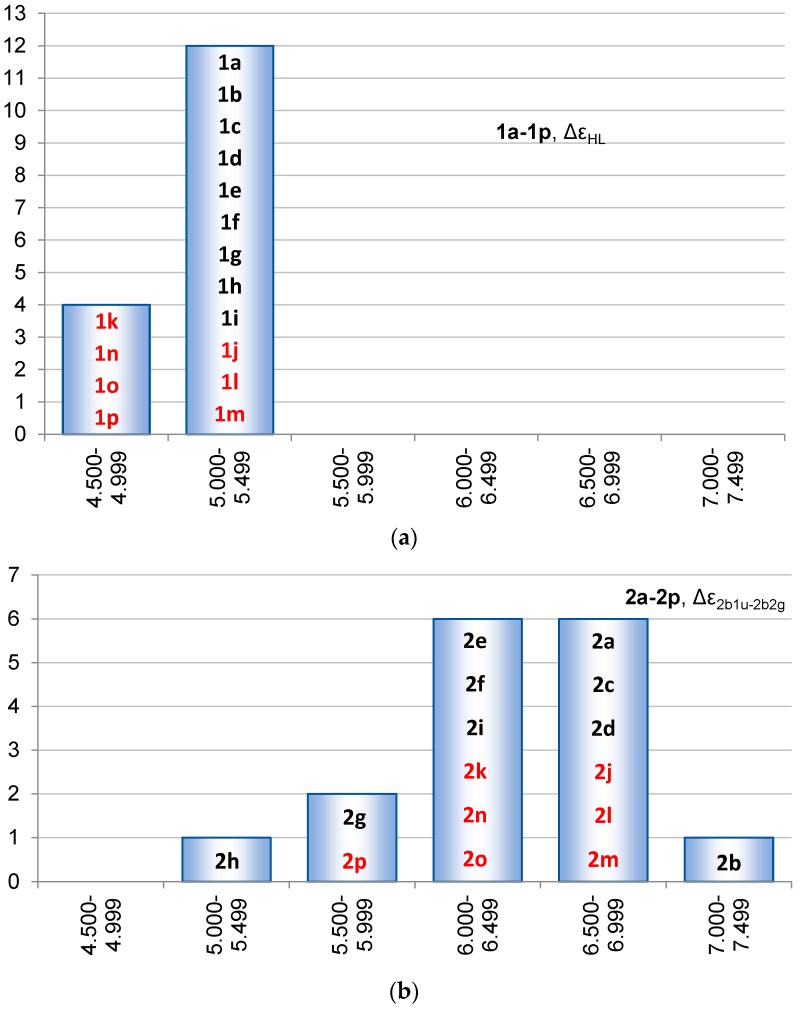
Distribution in (**a**) the HOMO-LUMO energy gaps (Δε_HL_) for **1a**–**1p** and (**b**) the 2b_1u_–2b_2g_ energy gaps for **2a**–**2p** (the Δε_HL_ for **2a**). Compound numbers of compounds substituted at the Si atoms in black and those substituted at the C atoms in red.

An interesting feature is the HOMO-LUMO gap (Δε_HL_) and its variation among the differently substituted siloles. As seen in Figure 11 the variation in Δε_HL_ is small because most siloles have Δε_HL_ in the range from −5.35 to −4.57 eV (ΔΔε_HL_ = 0.78 eV) with the smallest for **1k** and **1p**, and the largest for **1h**. Despite this, the separate spans in the HOMOs and LUMOs of **1a**–**1p** are 4.17 and 3.77 eV, i.e., the two orbitals are essentially affected to the same extents by the various substituents so that a small ΔΔε_HL_ results. 

For the π-symmetric MOs of the 1,4-disilacyclohexa-2,5-dienes the fragment represented by the two C=C double bonds contribute with a set of fragment orbitals which are analogous to the four π-MOs of *D*_2h_ symmetric cyclobutadiene. And the local π(SiR_2_) and π*(SiR_2_) orbitals combine into two b_1u_ and into two b_2g_ symmetric 2 × SiR_2_ fragment orbitals, respectively. In addition, from the shapes of the calculated orbitals it can be concluded that the two Si atoms contribute with 3d AOs in an a_u_ symmetric combination (Figure 7) contained in the 1a_u_ orbital which for several of the 1,4-disilacyclohexa-2,5-dienes is the LUMO. 

As there is a variation among **2a**–**2p** as to which orbital is HOMO and LUMO, we consider herein the two orbitals which correspond to HOMO and LUMO of **2a** (Figure 9 and Figure 10, and the Supporting Information). The HOMO of **2a** is the 2b_1u_ orbital and its LUMO is the 2b_2g_ orbital. For each of the 1,4-disilacyclohexa-2,5-diene considered here the first strongly allowed excitations involve transitions between these two orbitals, although they are not always HOMO and LUMO. For **2j**, **2k**, **2n**, **2o** and **2p** the 2b_1u_ orbital is HOMO-1, for **2b** and **2c** it is HOMO-2, for **2i** it is HOMO-4, while for the other eight 1,4-disilacyclohexa-2,5-dienes it is the HOMO. With regard to the 2b_2g_ orbital it is LUMO for **2a**–**2e**, **2i**–**2m** and **2p** and LUMO+1 for **2f**–**2h** and **2n**–**2o**. Yet, it is not possible to see any distinct pattern relating the characteristics of the substituents with the ordering of the orbitals of the 1,4-disilacyclohexa-2,5-dienes. 

On average the ε_2b1u_ of **2a**–**2p** is −7.64 eV, i.e., at a lower energy than that of siloles (ε_HOMO_ = −6.78 eV). The lowest ε_2b1u_ is found for **2b** (−9.16 eV), whereas **2p** is highest in energy (−5.45 eV). Thus, the span in the ε_2b1u_ among the various **2a**–**2p** is 3.71 eV, slightly lower than the corresponding span of the siloles (4.17 eV). If one regards the orbital energy variation through variation of the substituents at Si as in **2a**–**2i**, the ε_2b1u_ can be tuned within a larger interval (3.55 eV) than the ε_HOMO_ of the siloles (1.19 eV), a result that stems from the lack of contribution from the SiR_2_ segment to the HOMO of siloles. The two extremes for the siloles are **1f** (R = SiF_3_) and **1i** (R = NH_2_), and for 1,4-disilacyclohexa-2,5-dienes they are **2b** (R = F) and **2h** (R = SiMe_3_), respectively. 

The variation in orbital symmetries is large also for the LUMOs of **2a**–**2p**, and for that reason we analyze the 2b_2g_ orbitals of **2b**–**2p** that are analogous to LUMO of **2a**. First, the variation in the ε_2b2g_ with the substituents R at Si in **2a**–**2i** (2.12 eV) is larger than found for ε_LUMO_ in the corresponding siloles (1.32 eV). When regarding all 1,4-disilacyclohexa-2,5-dienes the average value of ε_2b2g_ is −1.28 eV, which is higher than for the siloles (−1.70 eV). The ε_2b2g_ ranges from −2.86 to 0.21 eV, so that the energy variation in this orbital among **2a**–**2p** (3.07 eV) is smaller than ε_LUMO_ among **1a**–**1p** (3.77 eV). Naturally, the electron withdrawing trifluorosilyl group gives ε_2b2g_ that is the lowest in energy (**2n**) and the electron donating amino group gives the highest (**2p**). Yet, the HOMO and LUMO of 1,4-disilacyclohexa-2,5-dienes do not respond similarly to the substitution leading to a factor 2.1 larger span in the Δε_HL_ for the 1,4-disilacyclohexa-2,5-dienes than for the siloles. 

In clear difference to the Δε_HL_ of siloles, the 1,4-disilacyclohexa-2,5-dienes have a large variation in the Δε_2b1u-2b2g_ (Δε_HL_ for **2a**) ranging from 5.49 to 7.15 eV. The two species with the largest and smallest Δε_2b1u-2b2g_ are **2b** and **2h**, respectively. Although the spread in Δε_2b1u-2b2g_ of **2a**–**2p** is distributed among the different energy intervals most 1,4-disilacyclohexa-2,5-dienes have Δε_2b1u-2b2g_ within the range 6.00–7.00 eV (Figure 11b). Still, the frontier orbital energies of 1,4-disilacyclohexa-2,5-dienes reveal a much stronger response to the choice of substituents than what is the case for the siloles.

### 2.3. Singlet State Excitation Energies

The goal is to identify means that can be used to qualitatively predict the excitation energies for the first allowed transitions of siloles and 1,4-disilacyclohexa-2,5-dienes. For all siloles **1a**–**1p** the lowest vertically excited singlet state is of B or B_2_ symmetry, depending on whether a particular silole is *C*_2_ or *C*_2v_ symmetric, and these transitions are dominated by the HOMO to LUMO excitation. Furthermore, the first transitions are allowed throughout the siloles. 

**Figure 12:**
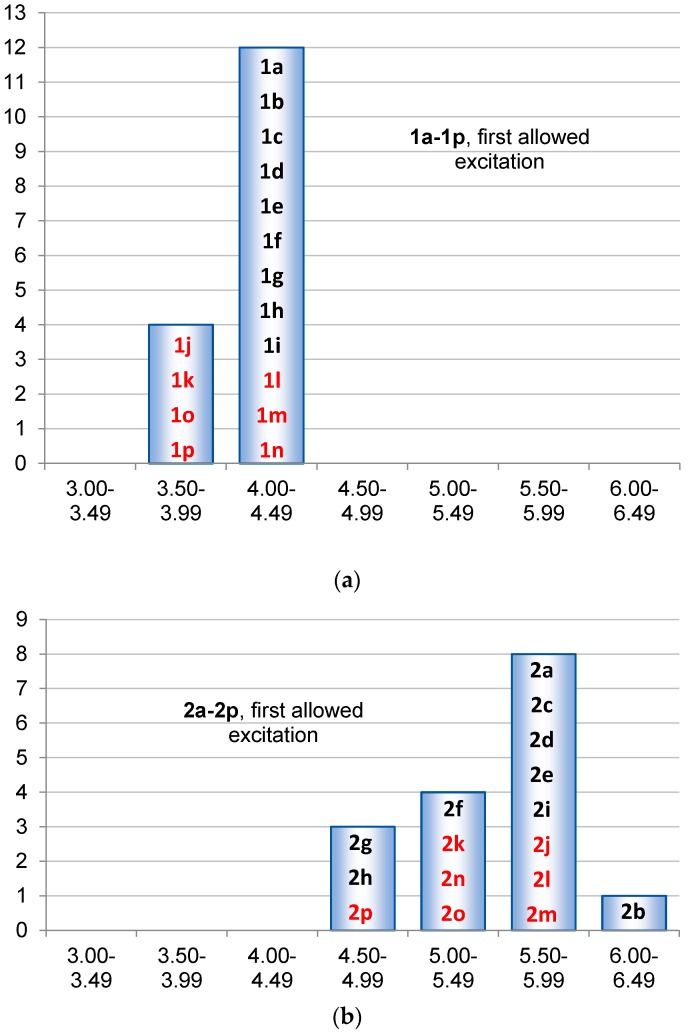
The distributions in the vertical excitation energies for the first allowed transitions to singlet excited states of (**a**) **1a**–**1p** and of (**b**) **2a**–**2p** calculated at TD-PBE0/6-31+G(2d,p)// PBE0/6-31G(d) level. Compound numbers of compounds substituted at the Si atoms in black and those substituted at the C atoms in red.

Most of the siloles also have a strong low-energy transition to a state of A or A_1_ symmetry. However, the excitation energies to the lowest singlet excited state of **1a**–**1p** are relatively similar (within 0.88 eV, Figure 12a), which is linked to the fact that Δε_HL_ of **1a**–**1p** display only a small variation with the substituents. As noted earlier, the substituents R at Si have only a small influence on the first excitation, and this is confirmed through our TD-DFT calculations because for **1a**–**1i** the first excitation energy varies within the interval 4.06–4.41 eV, i.e., a range of merely 0.35 eV. For **2a**–**2p** the first vertically excited singlet states are dark, or nearly dark, and distributed over a range of 2.13 eV, from 3.46 to 5.59 eV. The first allowed excitation for all 1,4-disilacyclohexa-2,5-dienes includes the transition from the 2b_1u_ to the 2b_2g_ orbitals which for **2a** are the HOMO and LUMO, respectively. Hence, we will consider here only the first allowed transitions (Figure 12). The first allowed excitations of **2a**–**2p** are distributed over a range of 1.52 eV, from 4.69 eV in **2h** to 6.21 eV in **2b**. The wide distribution in the lowest allowed excitation energies goes well with the wide distribution in the Δε_2b1u-2b2g_ observed above. Importantly, for the 1,4-disilacyclohexa-2,5-diene the substituents R at Si (**2a**–**2i**) also have a much larger impact on the lowest allowed excitation energy than for the siloles as the variation spans the same values as the variation of the entire set **2a**–**2p**, i.e., from 4.69 to 6.21 eV. As the corresponding siloles (**1a**–**1i**) only display a range of 0.35 eV, that of the 1,4-disilacyclohexa-2,5-dienes is a factor 4.3 larger.

Considering that electron withdrawing and electron donating groups can have opposing effects on HOMO and LUMO the usage of both as substituents at either the single Si atom in siloles or the two Si atoms in 1,4-disilacyclohexa-2,5-dienes could have intriguing results. For the silole having an SiF(SiMe_3_) moiety we calculate the lowest strongly allowed excitation at 4.01 eV, however, this is not a markedly shifted transition when compared to what is regular for siloles as seen in Figure 12a. In contrast, for the two 1,4-disilacyclohexa-2,5-diene isomers having two SiF(SiMe_3_) moieties we calculate a first strongly allowed transition at energies 4.44 (*E*-isomer) and 4.65 eV (*Z*-isomer), respectively, i.e., even lower than calculated for **2h** (4.69 eV). The low-lying transition of the *E*-isomer is particularly interesting and points to an additional means for influencing the excitation energies through choice of substituents. Clearly, as the R = SiMe_3_ substituents lead to a species (**2h**) with a high-lying occupied 2b_1u_ orbital while the R = F substituents lead to a species (**2b**) with a low-lying empty 2b_2g_ orbital, it is obvious that the 1,4-disilacyclohexa-2,5-diene having both fluoro and trimethylsilyl substituents at Si will have a small Δε_2b1u-2b2g_ gap (5.49 eV for *E*-isomer and 5.67 eV for *Z*-isomer) and a low-lying excitation for the transition involving these orbitals. Interestingly, a similar effect is not achieved with a 1,4-disilacyclohexa-2,5-diene having one SiF_2_ and one Si(SiMe_3_)_2_ moiety as this species has its first strongly allowed excitation at an energy of 4.81 eV. 

### 2.4. On Dimerization Reactions

An aspect that influences the utility of the substituted siloles and 1,4-disilacyclohexa-2,5-dienes is their various tendencies to undergo dimerization and further oligomerization reactions. It is well-established that siloles dimerize through [4 + 2] cycloaddition reactions when not properly substituted [37]. However, we now find that the activation barrier for this dimerization to a significant extent is influenced by the substituent at Si. Furthermore, this influence seems to be of electronic rather than of steric origin because the activation barrier for silole **1g**, having two sterically unencumbered, yet, σ-electron donating SiH_3_ substituents, is 7.6 kcal/mol higher than that of **1a** (Table 1). 

Conversely, for silole **1b** with electron withdrawing fluoro substituents at Si the activation energy is 3.0 kcal/mol lower than for **1a**. Indeed, the activation barrier for **1g** is sufficiently high to significantly hampered dimerization. 

In addition to the electronic effect exercised by the SiH_3_ group larger silyl groups will introduce steric congestion at the transition state, further raising the activation barrier. Thus, with moderate steric bulk imposed by silyl groups at the Si it should be possible to vary the substituents at the C atoms more widely than what is the case in siloles presently found in the field of organic electronics. 

With regard to the 1,4-disilacyclohexa-2,5-dienes, these have not been found to oligomerize to any detectable extent [21,23,25], a feature explained by the fact that their dimerization would involve a symmetry-forbidden [2 + 2] cycloaddition in contrast to the siloles which dimerize following a [4 + 2] cycloaddition path. 

### 2.5. Cyclobutadisiloles

**Figure 13:**
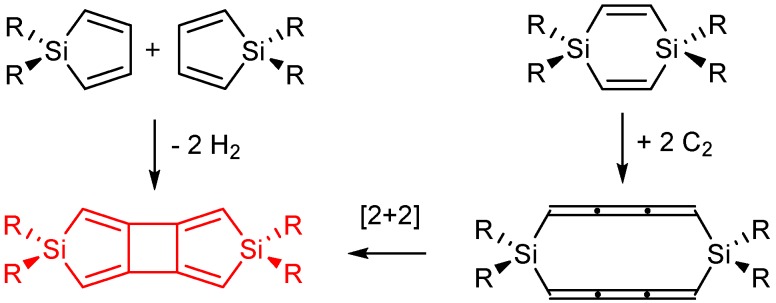
Formal relationship showing how siloles and 1,4-disilacyclohexa-2,5-dienes are linked with cyclobutadisiloles (in red).

**Figure 14:**
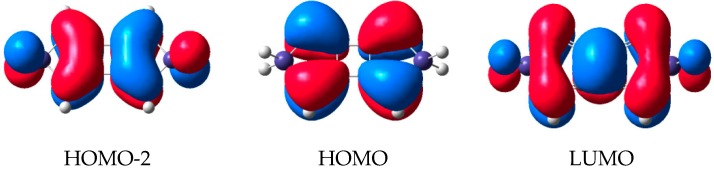
Frontier orbitals of unsubstituted cyclobutadisilole (R = H).

The variation of the substituents at the Si atom in siloles only leads to a modest change in the first excitation of 0.35 eV. The 1,4-disilacyclohexa-2,5-dienes, on the other hand, display a large response to this substitution but have much higher excitation energies for the first allowed transitions. Now, can the good features of siloles and 1,4-disilacyclohexa-2,5-dienes be combined, i.e., can one identify a compound class with large variation in the energies of their lowest strongly allowed excitations at the same time as they are at fairly low energies? Indeed, cyclobutadisiloles, or more fully cyclobuta[1,2-*c*:3,4-*c*’]disiloles, investigated by us earlier are those species [24]. They can be deduced formally through replacement of the two C=C double bonds with two C=C=C=C cumulene segments, providing diene-type fragment orbitals, followed by a collapse of their central C=C double bonds into a cyclobutane ring (Figure 13). The cyclobutadisiloles can also be regarded as silole dimers. Indeed, the HOMO of the parent cyclobutadisolole can be viewed as the out-of-phase combination of the HOMO of **1a**, and the LUMO can be seen as the in-phase combination of the corresponding orbital of **1a** (Figure 14). Yet, the first strongly allowed transition involves an excitation from HOMO-2 to LUMO, and these orbitals and the transition are analogous to what is found for 1,4-disilacyclohexa-2,5-dienes. 

Both the HOMO-2 and LUMO orbitals have large contributions from Si meaning that substituents at these atoms can have large effects on the excitation energies. Indeed, with R = H (**3a**) the transition is at 4.56 eV while with R = F (**3b**) it increases to 4.77 eV and with R = SiMe_3_ (**3h**) it decreases to 3.47 eV, i.e., a variation by 1.30 eV. An excitation energy of 3.47 eV corresponds to an absorption wavelength of 357 nm, i.e., nearly in the visible wavelength region. The variation in the first strong transition among the corresponding siloles (**1a**, **1b** and **1h**) and 1,4-disilacyclohexa-2,5-dienes (**2a**, **2b** and **2h**) are 0.29 and 1.52 eV, respectively. Thus, with the cyclobutadisiloles we have designed a compound (**3h**) with a first strongly allowed excitation which is lower by 0.94 and 1.22 eV than the analogous transitions in **1h** and **2h**, respectively. The further tailoring of the excitation energies can certainly be achieved through substituents at the C atoms, likely enabling design of cyclobutadisiloles with excitation energies well within the visible wavelength region. 

## 3. Computational Methods

All calculations were carried out with the Gaussian 09 program package [38]. The geometry optimizations were performed using the PBE0 hybrid density functional theory (DFT) level [39,40], with the 6-31G(d) valence double-zeta basis set of Pople and Hariharan [41]. The stationary points were subjected to frequency calculations to examine their characters as minima or saddle points. To evaluate vertical excitations to electronically excited states, time-dependent DFT (TDDFT) calculations were carried out on the DFT optimized geometrics. These TDDFT calculations were performed with the PBE0 functional using the 6-31+G(2d,p) valence double-zeta basis set [42,43,44].

## 4. Conclusions

Through DFT and TD-DFT computations of the geometrical, electronic, and optical properties we have revealed similarities and dissimilarities in the effects of substituents at siloles and 1,4-disilacyclohexa-2,5-dienes. The main differences are found in the impact substituents have on the electronic structure and the energies of the lowest electronically excited states. For siloles the lowest electronic transition is a HOMO-LUMO transition and it is strongly allowed. For 1,4-disilacyclohexa-2,5-dienes the first strongly allowed transition corresponds to the 2b_1u_ to 2b_2g_ transition which for some of the species is the HOMO to LUMO excitation. The HOMO-LUMO gap of the siloles can be varied in the range 4.5 to 5.4 eV, while for 1,4-disilacyclohexa-2,5-diene the 2b_1u_ to 2b_2g_ energy gap can be varied from 5.4 to 7.2 eV. As a consequence, the lowest strongly allowed electronic excitation varies with substituents to a much larger degree in 1,4-disilacyclohexa-2,5-dienes than in siloles. Thus, 1,4-disilacyclohexa-2,5-diene, or species derived based on them, could represent interesting new building blocks for molecular wires and materials for optoelectronics applications. In particular cyclobutadisiloles combine the good features of siloles and 1,4-disilacyclohexa-2,5-dienes as they display low-lying electronic transitions for which excitation energies can be varied extensively through substitution. Indeed, it should be possible to design cyclobutadisiloles with strongly allowed transitions in the visible wavelength region. 

## Figures and Tables

**Table 1:** Activation (∆G^‡^) and reaction (∆G) free energies (kcal/mol) for the dimerization of three selected siloles following a [4 + 2] cycloaddition path ^1^.

R =	H (1a)	F (1b)	SiH_3_ (1g)
∆G^‡^	21.5	18.6	29.2
∆G	−23.8	−30.4	−15.2

^1^ Calculated at PBE0/6-31G(d) level.

## Supplementary Materials

The following are available online. Figures S1 and S2 with frontier molecular orbitals of siloles and 1,4-disilacyclohexa-2,5-dienes, respectively, Tables S1–S4 with orbital energies and orbital energy gaps, Tables S5 and S6 with excitation energies of siloles, Tables S7 and S8 with excitation energies of 1,4-disilacyclohexa-2,5-dienes, Tables S9 and S10 with data on bond length and angles of siloles, Tables S11 and S12 with data on bond lengths and angles of 1,4-disilacyclohexa-2,5-dienes, and listings of Cartesian coordinates of all investigated compounds.
