# Supplementary material for: A Computational Investigation of the Substituent Effects on Geometric, Electronic, and Optical Properties of Siloles and 1,4-Disilacyclohexa-2,5-dienes
Source: Molecules. 2017 Feb 28;22(3):370. doi: 10.3390/molecules22030370 (PMC6155320; doi:10.3390/molecules22030370)
Supplement: Supplementary file 1 [file molecules-22-00370-s001.pdf]

Supporting Information
for
A computational investigation of the substituent effects on geometric, electronic, and optical properties of siloles and 1,4-disilacyclohexa-2,5-dienes

Aleksandra V. Denisova,[1] Julius Tibbelin,[2] Rikard Emanuelsson[3] and Henrik Ottosson*[1]

[1] Department of Chemistry – Ångström Laboratory, Uppsala University, Box 523, 751 20 Uppsala, Sweden.

[2] Department of Chemistry – BMC, Uppsala University, Box 576, 751 23 Uppsala, Sweden.

[3] Nanotechnology and Functional Materials, Department of Engineering Sciences, Uppsala University, Box 534, 751 21 Uppsala, Sweden

Contents

Molecular orbitals of siloles 2

Molecular orbitals of 1,4-disilacyclohexa-2,5-dienes 6

Orbital energies and HOMO-LUMO energy gaps of siloles 10

Orbital energies and HOMO – LUMO and HOMO-n – LUMO+m energy gaps of 1,4-disilacyclohexa-2,5-dienes 11

Electronic excitation energies of siloles 12

Electronic excitation energies of 1,4-disilacyclohexa-2,5-dienes 13

Bond lengths and angles in siloles 14

Bond lengths and angles in 1,4-disilacyclohexa-2,5-dienes 15

Cartesian coordinates and absolute energies 16

Figure S1: Frontier molecular orbitals of siloles

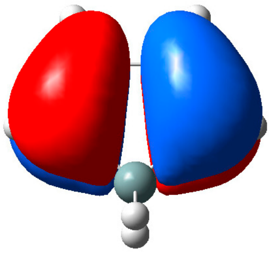

HOMO of **1a**
a_2 symmetry

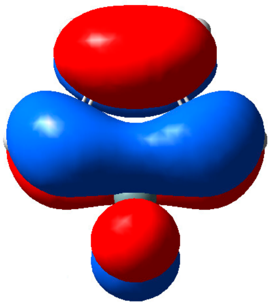

LUMO of **1a**
b_1 symmetry

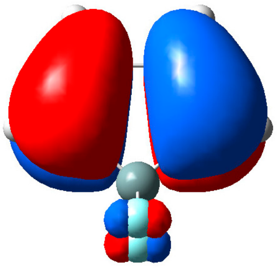

HOMO of **1b**
a_2 symmetry

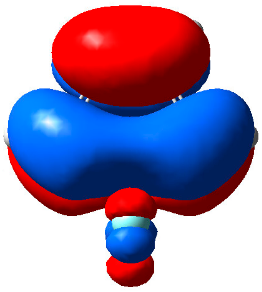

LUMO of **1b**
b_1 symmetry

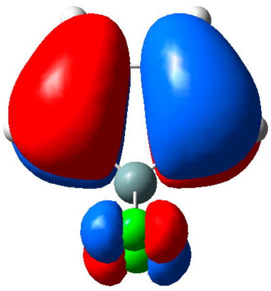

HOMO of **1c**
a_2 symmetry

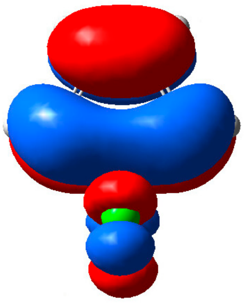

LUMO of **1c**
b_1 symmetry

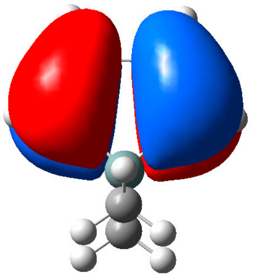

HOMO of **1d**
a_2 symmetry

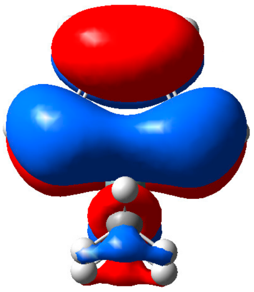

LUMO of **1d**
b_1 symmetry

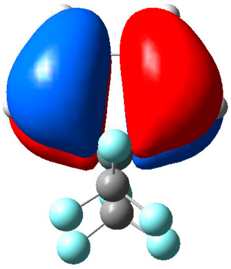

HOMO of **1e**
a_2 symmetry

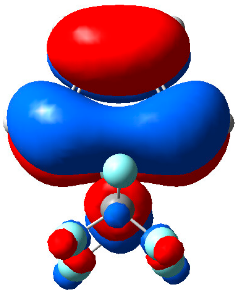

LUMO of **1e**
b_1 symmetry

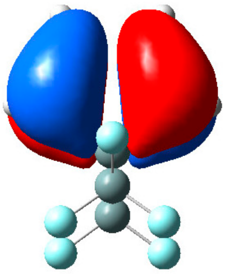

HOMO of **1f**
a_2 symmetry

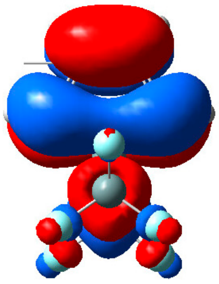

LUMO of **1f**
b1 symmetry

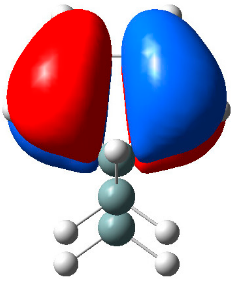

HOMO of **1g**
a2 symmetry

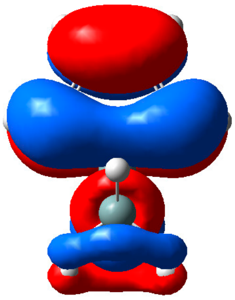

LUMO of **1g**
b_1 symmetry

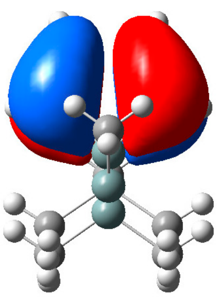

HOMO of **1h**
a_2 symmetry

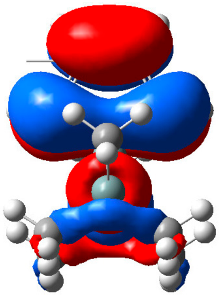

LUMO of **1h**
b_1 symmetry

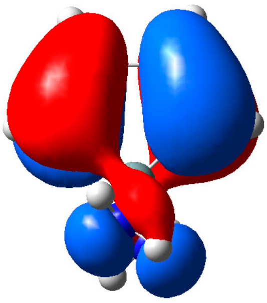

HOMO of **1i**
a_2 symmetry

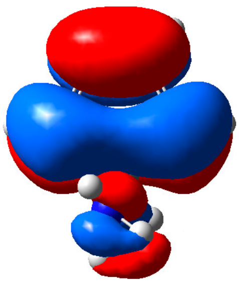

LUMO of **1i**
b_1 symmetry

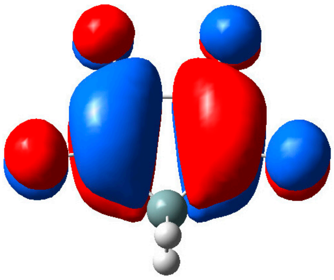

HOMO of **1j**
a_2 symmetry

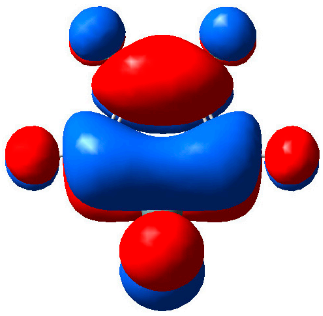

LUMO of **1j**
b_1 symmetry

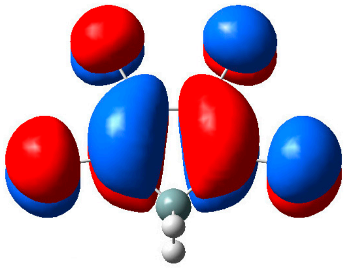

HOMO of **1k**
a_2 symmetry

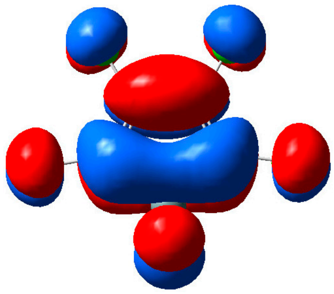

LUMO of **1k**
b_1 symmetry

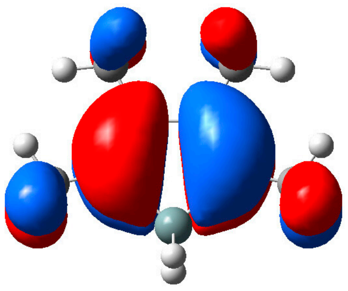

HOMO of **1l**
a_2 symmetry

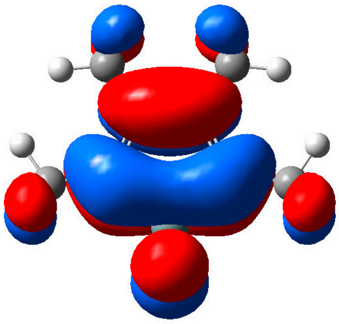

LUMO of **1l**
b_1 symmetry

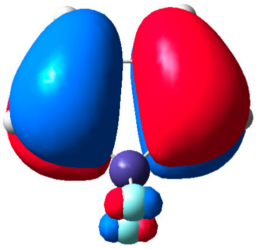

HOMO of **1m**
a_2 symmetry

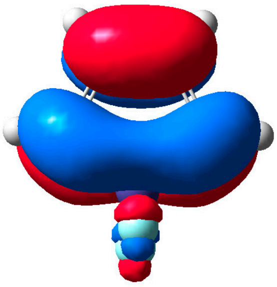

LUMO of **1m**
b_1 symmetry

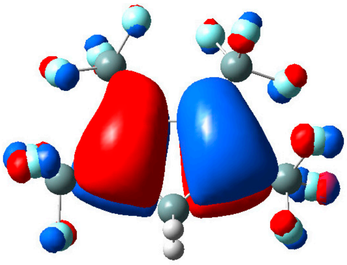

HOMO of **1n**
a_2 symmetry

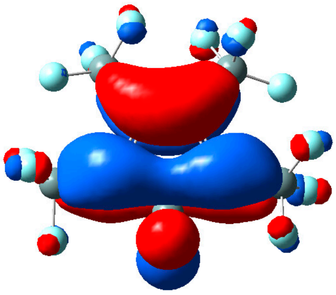

LUMO of **1n**
b_1 symmetry

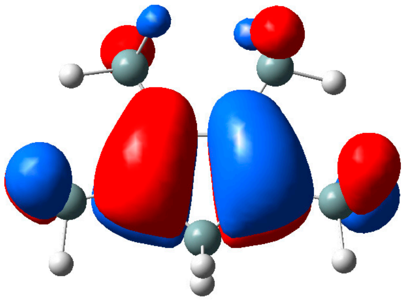

HOMO of **1o**
a_2 symmetry

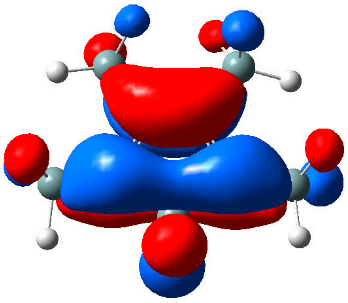

LUMO of **1o**
b_1 symmetry

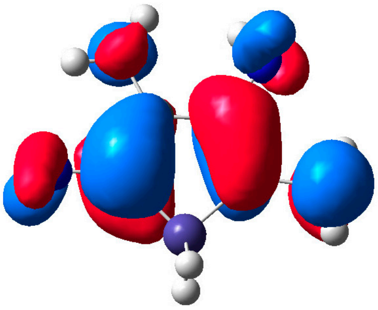

HOMO of **1p**
a_2 symmetry

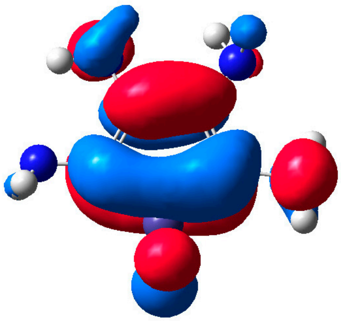

LUMO of **1p**
b_1 symmetry

Figure S2: Frontier molecular orbitals of 1,4-disilacyclohexa-2,5-dienes

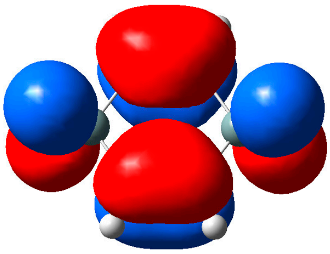

HOMO of **2a**
b_{1u} symmetry

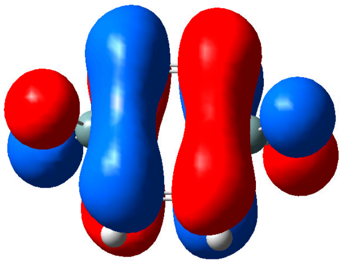

LUMO of **2a**
b_{2g} symmetry

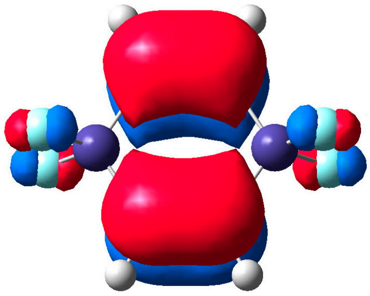

HOMO-2 of **2b**
b_{1u} symmetry

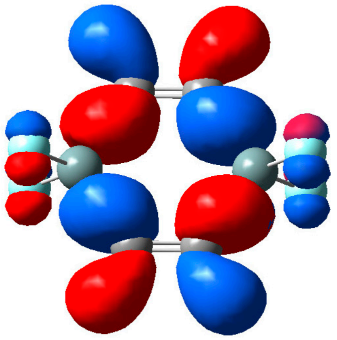

HOMO of **2b**

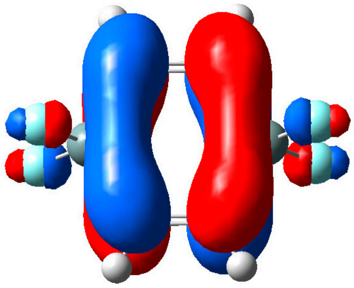

LUMO of **2b**
b_{2g} symmetry

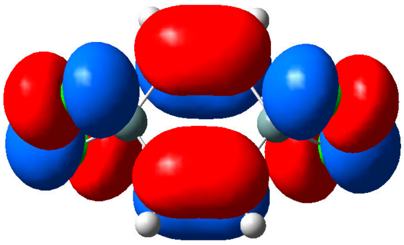

HOMO-2 of **2c**
b_{1u} symmetry

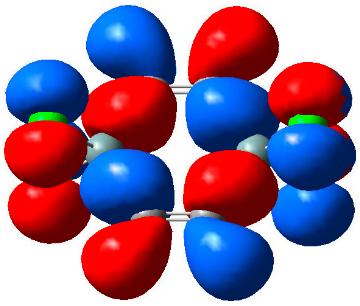

HOMO of **2c**

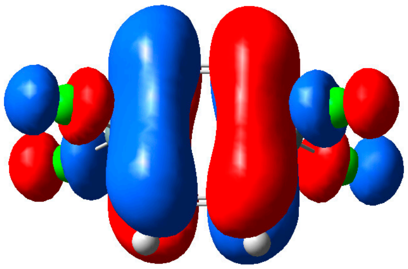

LUMO of **2c**
b_{2g} symmetry

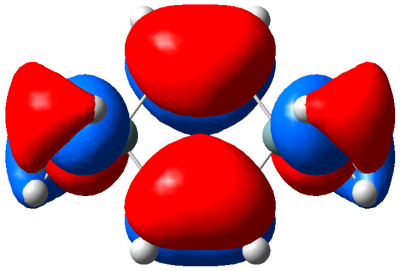

HOMO of **2d**
b_{1u} symmetry

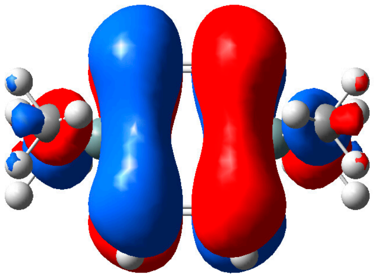

LUMO of **2d**
b_{2g} symmetry

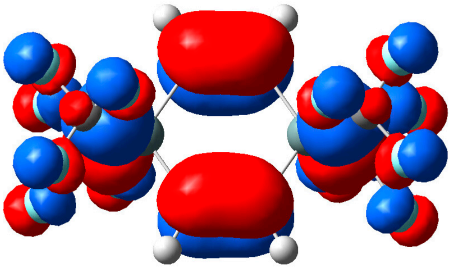

HOMO of **2e**
b_{1u} symmetry

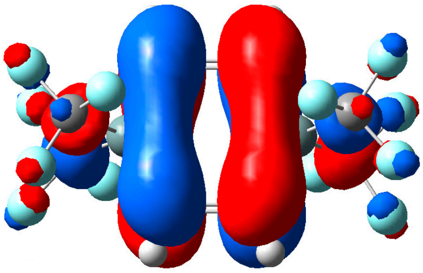

LUMO of **2e**
b_{2g} symmetry

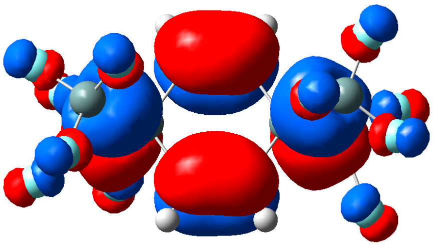

HOMO of **2f**
b_{1u} symmetry

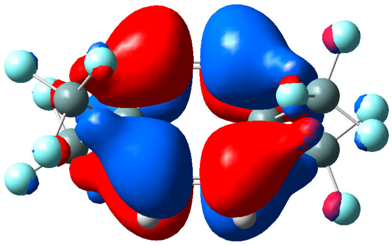

LUMO of **2f**
a_u symmetry

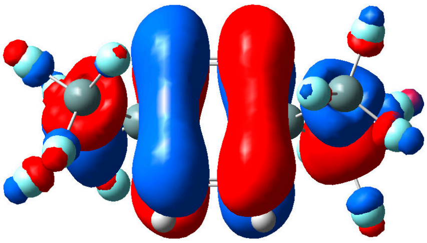

LUMO+1 of **2f**
b_{2g} symmetry

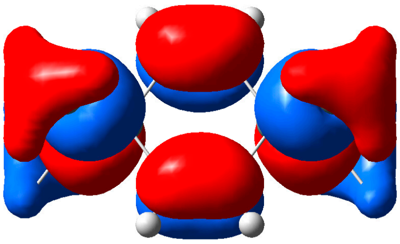

HOMO of **2g**
b_{1u} symmetry

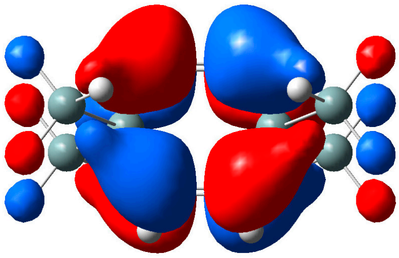

LUMO of **2g**
a_u symmetry

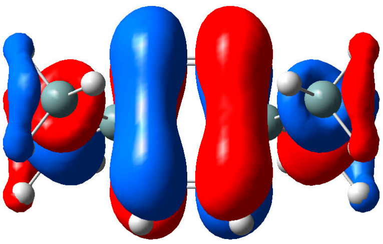

LUMO+1 of **2g**
b_{2g} symmetry

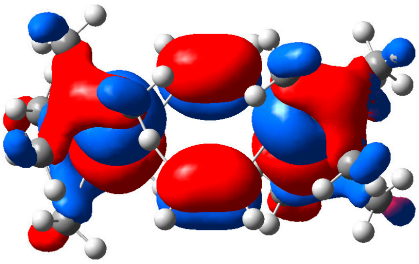

HOMO of **2h**
b_{1u} symmetry

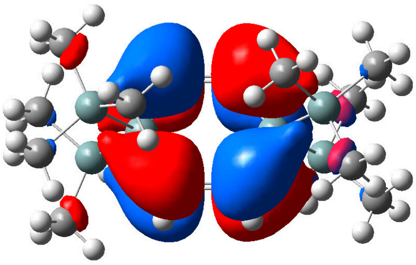

LUMO of **2h**
a_u symmetry

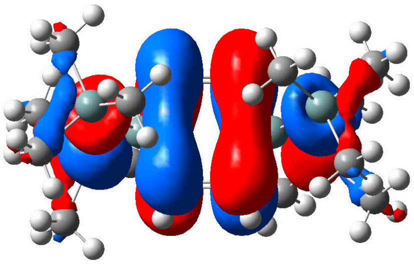

LUMO+1 of **2h**
b_{2g} symmetry

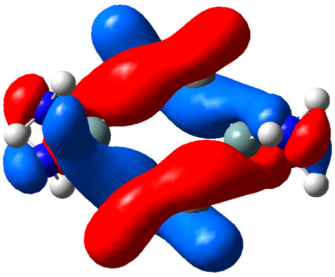

HOMO-4 of **2i**
b_{1u} symmetry

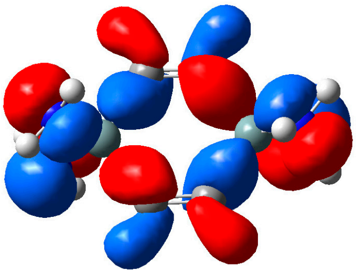

HOMO of **2i**

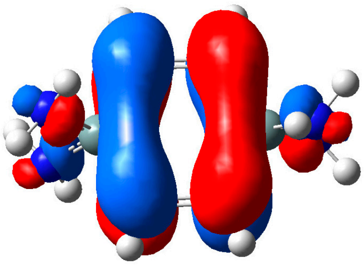

LUMO of **2i**
b_{2g} symmetry

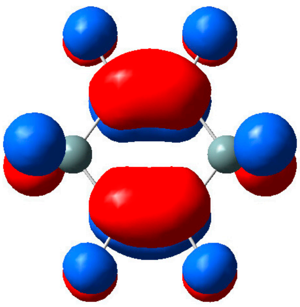

HOMO-1 of **2j**
b_{1u} symmetry

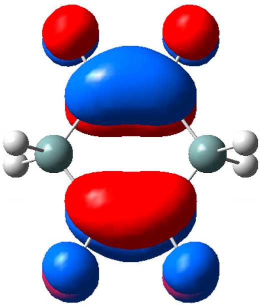

HOMO of **2j**
b_{3g} symmetry

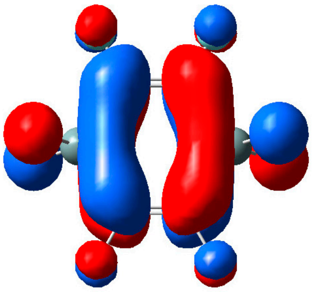

LUMO of **2j**
b_{2g} symmetry

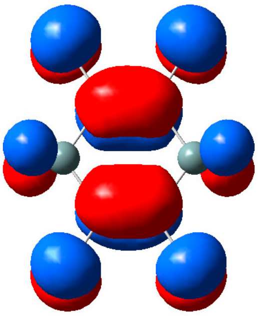

HOMO-1 of **2k**
b_{1u} symmetry

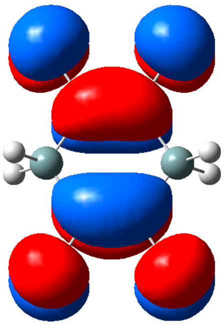

HOMO of **2k**
b_{3g} symmetry

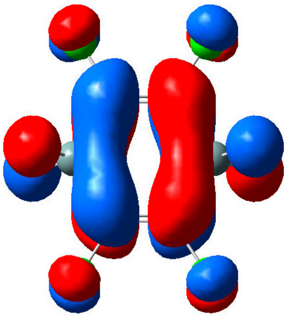

LUMO of **2k**
b_{2g} symmetry

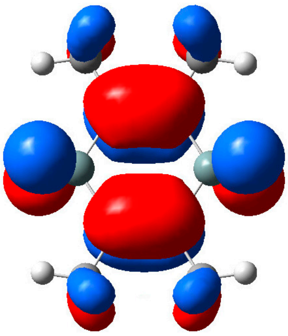

HOMO of **2l**
b_{1u} symmetry

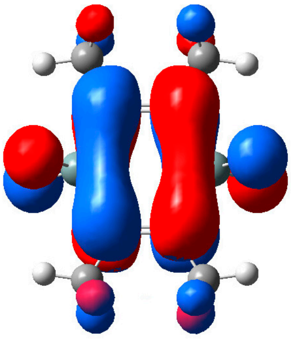

LUMO of **2l**
b_{2g} symmetry

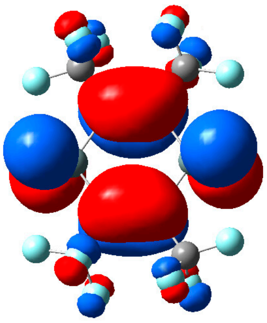

HOMO of **2m**
b_{1u} symmetry

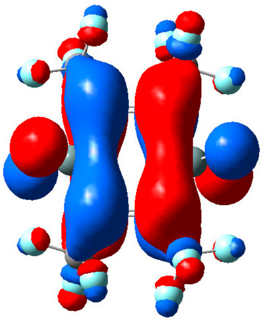

LUMO of **2m**
b_{2g} symmetry

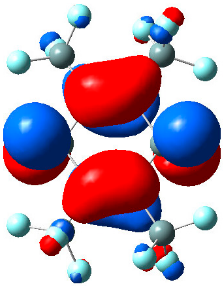

HOMO-1 of **2n**
b_{1u} symmetry

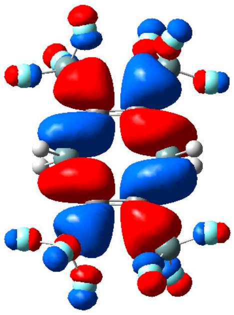

HOMO of **2n**

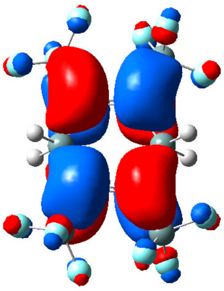

LUMO of **2n**
a_u symmetry

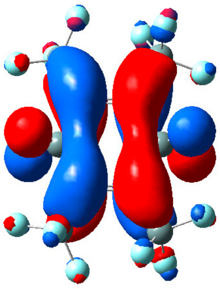

LUMO+1 of **2n**
b_{2g} symmetry

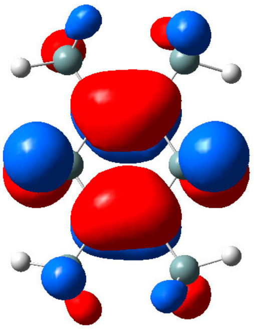

HOMO-1 of **2o**
b_{1u} symmetry

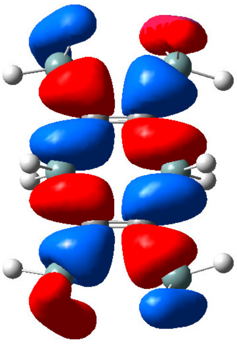

HOMO of **2o**

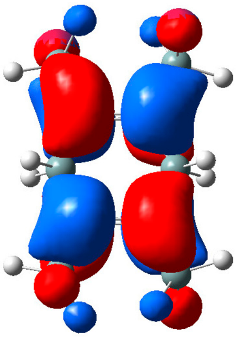

LUMO of **2o**
a_u symmetry

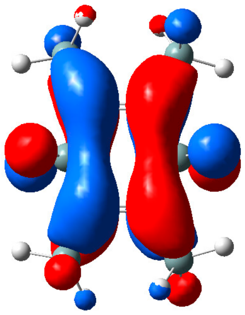

LUMO+1 of **2o**
b_{2g} symmetry

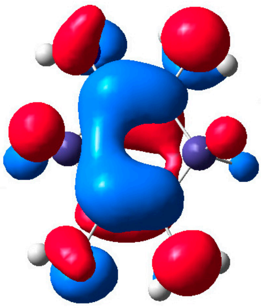

HOMO-1 of **2p**
b_{1u} symmetry

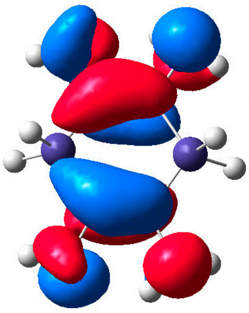

HOMO of **2p**
b_{3g} symmetry

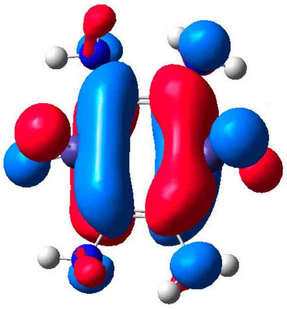

LUMO of **2p**
b_{2g} symmetry

Orbital energies and HOMO-LUMO energy gaps of siloles

Table S1. Orbital energies and HOMO-LUMO energy gaps of siloles **1a – 1i** (in eV, calculated at PBE0/6-31G(d) level).

	**1a**	**1b**	**1c**	**1d**	**1e**	**1f**	**1g**	**1h**	**1i**
E_{LUMO}	-6.56	-7.06	-7.18	-6.24	-7.29	-7.33	-6.62	-6.14	-6.14
E_{HOMO}	-1.25	-1.92	-2.10	-0.94	-2.11	-2.01	-1.33	-0.79	-0.99
$\Delta E_{HOMO\text{-}LUMO}$	5.32	5.14	5.08	5.30	5.18	5.32	5.29	5.35	5.15

Table S2. Orbital energies and HOMO-LUMO energy gaps of siloles **1j – 1p** (in eV, calculated at PBE0/6-31G(d) level).

	**1j**	**1k**	**1l**	**1m**	**1n**	**1o**	**1p**
E_{LUMO}	-6.58	-6.78	-5.74	-8.44	-8.75	-7.09	-4.58
E_{HOMO}	-1.48	-2.22	-0.65	-3.25	-3.78	-2.31	-0.01
$\Delta E_{HOMO\text{-}LUMO}$	5.10	4.57	5.09	5.19	4.98	4.78	4.57

Orbital energies and HOMO – LUMO and HOMO-n – LUMO+m energy gaps of 1,4-disilacyclohexa-2,5-dienes

Table S3. Orbital energies and HOMO-LUMO and HOMO-n – LUMO+m energy gaps of 1,4-disilacyclohexa-2,5-dienes **2a – 2i** (in eV, calculated at PBE0/6-31G(d) level).

	**2a**	**2b**	**2c**	**2d**	**2e**	**2f**	**2g**	**2h**	**2i**
E_{LUMO+m}	-	-9.16	-8.85	-	-	-	-	-	-7.01
E_{LUMO}	-7.57	-8.49	-8.59	-7.07	-8.59	-8.06	-6.70	-5.61	-6.40
E_{HOMO}	-0.73	-2.02	-2.24	-0.33	-2.17	-1.96	-1.03	-0.30	-0.52
E_{HOMO-n}	-	-	-	-	-	-1.95	-0.94	-0.12	-
$\Delta E_{HOMO-LUMO}$	6.84	6.47	6.35	6.74	6.42	6.10	5.67	5.32	5.88
$\Delta E_{HOMO-n-LUMO+m}$	-	7.15	6.60	-	-	6.11	5.76	5.49	6.49
	-	n = 2	n = 2	-	-	m = 1	m = 1	m = 1	n = 4

Table S4. Orbital energies and HOMO-LUMO and HOMO-n – LUMO+m energy gaps of 1,4-disilacyclohexa-2,5-dienes **2j – 2p** (in eV, calculated at PBE0/6-31G(d) level).

	**2j**	**2k**	**2l**	**2m**	**2n**	**2o**	**2p**
E_{LUMO+m}	-7.67	-7.78			-9.13	-7.81	-5.45
E_{LUMO}	-7.54	-7.64	-6.85	-8.89	-8.28	-6.82	-5.12
E_{HOMO}	-1.00	-1.57	-0.33	-2.33	-2.90	-1.60	0.21
E_{HOMO-n}					-2.86	-1.59	
$\Delta E_{HOMO-LUMO}$	6.54	6.07	6.52	6.56	5.38	5.22	5.32
$\Delta E_{HOMO-n-LUMO+m}$	6.67	6.21			6.27	6.22	5.66
	n = 1	n = 1	-	-	m = 1<br>n = 1	m = 1<br>n = 1	n = 1

Electronic excitation energies of siloles

Table S5. Electronic excitation energies of siloles **1a – 1i** (in eV, calculated at PBE0/6-31G(d) level).

Electronic state	**1a**	**1b**	**1c**	**1d**	**1e**	**1f**	**1g**	**1h**	**1i**
S_1	**4.40**	**4.12**	**4.06**	**4.35**	**4.18**	**4.35**	**4.34**	**4.41**	**4.13**
S_2	5.68	5.19	4.98	5.44	5.64	5.20	5.18	4.76	4.51
S_3	5.94	6.32	5.77	5.67	5.75	5.27	5.39	5.09	4.90
S_4	5.99	6.33	5.85	5.68	5.97	5.74	5.54	5.46	5.56
S_5	6.10	6.61	6.12	6.03	6.40	5.74	5.57	5.49	5.94

Table S6. Electronic excitation energies of siloles **1j – 1p** (in eV, calculated at PBE0/6-31G(d) level).

Electronic state	**1j**	**1k**	**1l**	**1m**	**1n**	**1o**	**1p**
S_1	**4.20**	**3.71**	**4.14**	**4.36**	**4.19**	**3.95**	**3.60**
S_2	4.87	4.65	5.08	5.21	4.29	4.12	3.77
S_3	5.19	5.05	5.21	5.52	4.33	4.22	3.95
S_4	5.93	5.24	5.57	5.91	5.58	5.43	4.45
S_5	6.37	5.32	5.73	6.79	5.99	5.73	4.90

First allowed excitations are marked in bold.

Electronic excitation energies of 1,4-disilacyclohexa-2,5-dienes

Table S7. Electronic excitation energies of 1,4-disilacyclohexa-2,5-dienes **2a – 2i** (in eV, calculated at PBE0/6-31G(d) level).

Electronic state	**2a**	**2b**	**2c**	**2d**	**2e**	**2f**	**2g**	**2h**	**2i**
S_1	5.53	4.90	4.92	5.29	5.32	4.86	4.43	4.13	4.55
S_2	5.72	5.57	5.37	5.64	**5.65**	**5.35**	**4.92**	**4.69**	5.00
S_3	5.87	5.97	**5.72**	**5.78**	5.78	5.45	5.32	4.85	5.35
S_4	**5.94**	**6.21**	5.82	5.92	5.79	5.62	5.46	5.08	5.37
S_5	6.24	6.99	6.05	6.13	6.02	5.64	5.48	5.19	5.44
S_6									**5.61**

Table S8. Electronic excitation energies of 1,4-disilacyclohexa-2,5-dienes **2j – 2p** (in eV, calculated at PBE0/6-31G(d) level).

Electronic state	**2j**	**2k**	**2l**	**2m**	**2n**	**2o**	**2p**
S_1	5.59	5.10	5.56	5.37	3.78	3.63	4.27
S_2	5.83	**5.29**	**5.58**	5.44	3.93	3.75	4.34
S_3	**5.83**	5.30	5.61	5.48	4.98	4.97	4.46
S_4	5.84	5.44	5.74	**5.69**	4.99	5.01	**4.78**
S_5	6.27	5.56	5.81	6.04	5.24	5.17	4.79
S_6					**5.43**	**5.31**	

First allowed excitations are marked in bold.

Bond lengths and angles in siloles

Table S9. Bond lengths and angles in siloles **1a – 1i** (optimized at PBE0/6-31G(d) level).

	**1a**	**1b**	**1c**	**1d**	**1e**	**1f**	**1g**	**1h**	**1i**
Si-C bond length, Å	1.8707	1.8558	1.8571	1.8792	1.8561	1.8632	1.8710	1.8775	1.8821
C=C bond length, Å	1.3475	1.3440	1.3447	1.3476	1.3477	1.3509	1.3517	1.3536	1.3454
C-C bond length, Å	1.4813	1.4948	1.4908	1.4818	1.4856	1.4738	1.4716	1.4680	1.4880
Angle R-Si-R, °	107.7660	105.2960	107.8323	110.2449	106.1945	113.7669	112.1668	117.9339	105.1581

Table S10. Bond lengths and angles in siloles **1j – 1q** (optimized at PBE0/6-31G(d) level).

	**1j**	**1k**	**1l**	**1m**	**1n**	**1o**	**1p**
Si-C bond length, Å	1.8827	1.8744	1.8673	1.8727	1.8824	1.8741	1.8906
C=C bond length, Å	1.3406	1.3488	1.3555	1.3490	1.3612	1.3646	1.3587
C-C bond length, Å	1.4813	1.4939	1.5071	1.5069	1.5019	1.5074	1.4800
Angle R-Si-R, °	110.6397	110.9590	106.7955	111.6681	110.3776	108.0016	105.9039

Bond lengths and angles in 1,4-disilacyclohexa-2,5-dienes

Table S11. Bond lengths and angles in 1,4-disilacyclohexa-2,5-dienes **2a** – **2i**.

	**2a**	**2b**	**2c**	**2d**	**2e**	**2f**	**2g**	**2h**	**2i**
Si-C bond length, Å	1.8713	1.8534	1.8574	1.8762	1.8603	1.8705	1.8764	1.8821	1.8744
C=C bond length, Å	1.3456	1.3457	1.3453	1.3469	1.3460	1.3452	1.3469	1.3485	1.3475
Angle R-Si-R, °	106.1040	105.4189	107.5479	108.6153	105.4290	105.6902	108.1325	110.8760	103.6802

Table S12. Bond lengths and angles in 1,4-disilacyclohexa-2,5-dienes **2j – 2q**.

	**2j**	**2k**	**2l**	**2m**	**2n**	**2o**	**2p**
Si-C bond length, Å	1.8769	1.8793	1.8715	1.8920	1.8912	1.8821	1.8711
C=C bond length, Å	1.3393	1.3413	1.3526	1.3442	1.3601	1.3606	1.3596
Angle R-Si-R, °	108.8446	109.7295	104.8629	110.9202	108.6589	106.2526	105.4112

Cartesian coordinates and absolute energies

1a:

PBE1PBE/6-31G(d): -445.15015 a.u.

Point group: C_{2v}

Cartesian coordinates:

C	0.00000000	1.35441100	-0.08899000
C	0.00000000	0.74065100	-1.28858500
C	0.00000000	-0.74065100	-1.28858500
C	0.00000000	-1.35441100	-0.08899000
H	0.00000000	2.43427600	0.01363700
H	0.00000000	1.27523400	-2.23839900
H	0.00000000	-1.27523400	-2.23839900
H	0.00000000	-2.43427600	0.01363700
Si	0.00000000	0.00000000	1.20133500
H	-1.20539200	0.00000000	2.08087000
H	1.20539200	0.00000000	2.08087000

1b:

PBE1PBE/6-31G(d): -643.58748 a.u.

Point group: C_{2v}

Cartesian coordinates:

C	0.00000000	1.36818200	-0.65412400
C	0.00000000	0.74740000	-1.84613300
C	0.00000000	-0.74740000	-1.84613300
C	0.00000000	-1.36818200	-0.65412400
H	0.00000000	2.44664600	-0.54850700
H	0.00000000	1.27187100	-2.80044800
H	0.00000000	-1.27187100	-2.80044800
H	0.00000000	-2.44664600	-0.54850700
Si	0.00000000	0.00000000	0.59969700
F	1.27460100	0.00000000	1.57251300
F	-1.27460100	0.00000000	1.57251300

1c:

PBE1PBE/6-31G(d): -1364.13695 a.u.

Point group: C_{2v}

Cartesian coordinates:

C	0.00000000	1.36483800	-1.09635900
C	0.00000000	0.74540800	-2.28985100
C	0.00000000	-0.74540800	-2.28985100
C	0.00000000	-1.36483800	-1.09635900
H	0.00000000	2.44160200	-0.97512800
H	0.00000000	1.27417000	-3.24192000
H	0.00000000	-1.27417000	-3.24192000
H	0.00000000	-2.44160200	-0.97512800
Si	0.00000000	0.00000000	0.16294900
Cl	-1.66462800	0.00000000	1.37609800
Cl	1.66462800	0.00000000	1.37609800

1d:

PBE1PBE/6-31G(d): -523.70931 a.u.

Point group: C_{2v}

Cartesian coordinates:

C	0.00000000	1.35041800	-0.73400900
C	0.00000000	0.74089000	-1.93583500
C	0.00000000	-0.74089000	-1.93583500
C	0.00000000	-1.35041800	-0.73400900
H	0.00000000	2.43239700	-0.63989600
H	0.00000000	1.27451600	-2.88683400
H	0.00000000	-1.27451600	-2.88683400
H	0.00000000	-2.43239700	-0.63989600
Si	0.00000000	0.00000000	0.57280700
C	-1.54716400	0.00000000	1.65122200
H	-1.57555500	-0.88519800	2.29672000
H	-1.57555500	0.88519800	2.29672000
H	-2.45210100	0.00000000	1.03536800
C	1.54716400	0.00000000	1.65122200
H	1.57555500	0.88519800	2.29672000
H	1.57555500	-0.88519800	2.29672000

H 2.45210100 0.00000000 1.03536800

1e:

PBE1PBE/6-31G(d): -1118.59751 a.u.

Point group: C_2

Cartesian coordinates:

C -1.36229200 -0.04530300 1.61761800
C -0.74241200 -0.02418400 2.81408700
C 0.74241200 0.02418400 2.81408700
C 1.36229200 0.04530300 1.61761800
H -2.43980800 -0.07892300 1.50668000
H -1.27324100 -0.04003600 3.76505400
H 1.27324100 0.04003600 3.76505400
H 2.43980800 0.07892300 1.50668000
Si 0.00000000 0.00000000 0.35776000
C -0.06526000 1.54089400 -0.80032800
C 0.06526000 -1.54089400 -0.80032800
F 0.95699500 1.55372800 -1.67662800
F 0.00000000 2.68372400 -0.09294800
F -1.20532600 1.57930800 -1.51534700
F -0.95699500 -1.55372800 -1.67662800
F 0.00000000 -2.68372400 -0.09294800
F 1.20532600 -1.57930800 -1.51534700

1f:

PBE1PBE/6-31G(d): -1621.57674 a.u.

Point group: C_{2v}

Cartesian coordinates:

C 0.00000000 1.35680100 1.81050500
C 0.00000000 0.73689500 3.01075800
C 0.00000000 -0.73689500 3.01075800
C 0.00000000 -1.35680100 1.81050500
H 0.00000000 2.43485100 1.69571700
H 0.00000000 1.27432100 3.95823700
H 0.00000000 -1.27432100 3.95823700
H 0.00000000 -2.43485100 1.69571700
Si 0.00000000 0.00000000 0.53351000
Si -1.93872300 0.00000000 -0.73112700
Si 1.93872300 0.00000000 -0.73112700
F -2.04133500 -1.28389800 -1.67120400
F -2.04133500 1.28389800 -1.67120400
F -3.21554500 0.00000000 0.22237200
F 3.21554500 0.00000000 0.22237200
F 2.04133500 1.28389800 -1.67120400
F 2.04133500 -1.28389800 -1.67120400

1g:

PBE1PBE/6-31G(d): -1026.26938 a.u.

Point group: C_{2v}

Cartesian coordinates:

C 0.00000000 1.35019100 -1.18236000
C 0.00000000 0.73578700 -2.38632600
C 0.00000000 -0.73578700 -2.38632600
C 0.00000000 -1.35019100 -1.18236000
H 0.00000000 2.42955800 -1.07306800
H 0.00000000 1.27425100 -3.33399400
H 0.00000000 -1.27425100 -3.33399400
H 0.00000000 -2.42955800 -1.07306800
Si 0.00000000 0.00000000 0.11286700
Si 1.94444400 0.00000000 1.42029700
H 1.99058500 -1.20673600 2.29662300
H 3.15572600 0.00000000 0.55170400
H 1.99058500 1.20673600 2.29662300
Si -1.94444400 0.00000000 1.42029700
H -1.99058500 -1.20673600 2.29662300
H -1.99058500 1.20673600 2.29662300
H -3.15572600 0.00000000 0.55170400

1h:

PBE1PBE/6-31G(d): -1261.93655 a.u.

Point group: C_{2v}

Cartesian coordinates:

C 0.00000000 1.34355200 1.81700700
C 0.00000000 0.73401300 3.02560900

C 0.00000000 -0.73401300 3.02560900
C 0.00000000 -1.34355200 1.81700700
H 0.00000000 2.42490600 1.71490700
H 0.00000000 1.27403200 3.97311600
H 0.00000000 -1.27403200 3.97311600
H 0.00000000 -2.42490600 1.71490700
Si 0.00000000 0.00000000 0.50556800
Si -2.02050000 0.00000000 -0.71005900
Si 2.02050000 0.00000000 -0.71005900
C 2.13533000 1.54156400 -1.80356500
H 3.09270800 1.55965200 -2.33917300
H 1.33516900 1.56984800 -2.55175300
H 2.06950600 2.45936500 -1.20885300
C 2.13533000 -1.54156400 -1.80356500
H 1.33516900 -1.56984800 -2.55175300
H 3.09270800 -1.55965200 -2.33917300
H 2.06950600 -2.45936500 -1.20885300
C 3.45187000 0.00000000 0.52518100
H 4.41840800 0.00000000 0.00626400
H 3.41209100 0.88326100 1.17156000
H 3.41209100 -0.88326100 1.17156000
C -2.13533000 1.54156400 -1.80356500
H -1.33516900 1.56984800 -2.55175300
H -3.09270800 1.55965200 -2.33917300
H -2.06950600 2.45936500 -1.20885300
C -2.13533000 -1.54156400 -1.80356500
H -3.09270800 -1.55965200 -2.33917300
H -1.33516900 -1.56984800 -2.55175300
H -2.06950600 -2.45936500 -1.20885300
C -3.45187000 0.00000000 0.52518100
H -3.41209100 0.88326100 1.17156000
H -4.41840800 0.00000000 0.00626400
H -3.41209100 -0.88326100 1.17156000

1i:

PBE1PBE/6-31G(d): -555.80032 a.u.

Point group: C_2

Cartesian coordinates:

C 0.00000000 1.35214300 -0.71474800
C -0.00545500 0.74396000 -1.91483700
C 0.00545500 -0.74396000 -1.91483700
C 0.00000000 -1.35214300 -0.71474800
H -0.00790500 2.43414400 -0.61880700
H -0.01586600 1.27360200 -2.86797100
H 0.01586600 -1.27360200 -2.86797100
H 0.00790500 -2.43414400 -0.61880700
Si 0.00000000 0.00000000 0.59448500
N -1.34665600 -0.26261500 1.64427200
H -1.58001600 0.44658400 2.32693000
H -2.17838500 -0.69548100 1.26606500
N 1.34665600 0.26261500 1.64427200
H 2.17838500 0.69548100 1.26606500
H 1.58001600 -0.44658400 2.32693000

1j:

PBE1PBE/6-31G(d): -841.70301 a.u.

Point group: C_{2v}

Cartesian coordinates:

C 0.00000000 1.33852100 0.41179700
C 0.00000000 0.74062400 -0.78813600
C 0.00000000 -0.74062400 -0.78813600
C 0.00000000 -1.33852100 0.41179700
Si 0.00000000 0.00000000 1.73571300
H -1.22040700 0.00000000 2.58013700
H 1.22040700 0.00000000 2.58013700
F 0.00000000 -2.66323600 0.58479600
F 0.00000000 -1.34119300 -1.97058500
F 0.00000000 1.34119300 -1.97058500
F 0.00000000 2.66323600 0.58479600

1k:

PBE1PBE/6-31G(d): -2282.89523 a.u.

Point group: C_{2v}

Cartesian coordinates:

C 0.00000000 1.33810800 0.69318200
C 0.00000000 0.74693400 -0.51916700
C 0.00000000 -0.74693400 -0.51916700
C 0.00000000 -1.33810800 0.69318200
Si 0.00000000 0.00000000 2.00576000
H -1.22256100 0.00000000 2.84664700
H 1.22256100 0.00000000 2.84664700
Cl 0.00000000 -3.03072900 0.95325300
Cl 0.00000000 -1.59684600 -2.00802100
Cl 0.00000000 1.59684600 -2.00802100
Cl 0.00000000 3.03072900 0.95325300

1l:

PBE1PBE/6-31G(d): -602.22836 a.u.

Point group: C_{2v}

Cartesian coordinates:

C 0.00000000 1.36697300 0.45542100
C 0.00000000 0.75357200 -0.75336700
C 0.00000000 -0.75357200 -0.75336700
C 0.00000000 -1.36697300 0.45542100
Si 0.00000000 0.00000000 1.72748700
H -1.20072100 0.00000000 2.61929500
H 1.20072100 0.00000000 2.61929500
C 0.00000000 -2.83715700 0.73189800
H 0.87834300 -3.12735900 1.32363200
H -0.87834300 -3.12735900 1.32363200
H 0.00000000 -3.45073400 -0.17493000
C 0.00000000 -1.46060000 -2.07855000
H 0.87994100 -1.19024500 -2.67569300
H 0.00000000 -2.54647600 -1.96505800
H -0.87994100 -1.19024500 -2.67569300
C 0.00000000 1.46060000 -2.07855000
H -0.87994100 1.19024500 -2.67569300
H 0.00000000 2.54647600 -1.96505800
H 0.87994100 1.19024500 -2.67569300
C 0.00000000 2.83715700 0.73189800
H -0.87834300 3.12735900 1.32363200
H 0.87834300 3.12735900 1.32363200
H 0.00000000 3.45073400 -0.17493000

1m:

PBE1PBE/6-31G(d): -1792.04316 a.u.

Point group: C_2

Cartesian coordinates:

C 0.00438100 1.33218500 0.85851500
C -0.02347400 0.75308700 -0.35957300
C 0.02347400 -0.75308700 -0.35957300
C -0.00438100 -1.33218500 0.85851500
Si 0.00000000 0.00000000 2.17468600
H -1.22619000 0.03471800 3.00727200
H 1.22619000 -0.03471800 3.00727200
C 0.00000000 -2.78845600 1.21938600
C 0.08414800 -1.54379300 -1.65783000
C -0.08414800 1.54379300 -1.65783000
C 0.00000000 2.78845600 1.21938600
F 0.89862300 -0.95278700 -2.53966800
F 0.55908200 -2.77434500 -1.45675000
F -1.12706100 -1.65896300 -2.20889400
F -0.89862300 0.95278700 -2.53966800
F -0.55908200 2.77434500 -1.45675000
F 1.12706100 1.65896300 -2.20889400
F -1.19345500 3.36048700 1.03378400
F 0.92188000 3.50127200 0.56723300
F 0.28181400 2.89674800 2.53839900
F -0.92188000 -3.50127200 0.56723300
F 1.19345500 -3.36048700 1.03378400
F -0.28181400 -2.89674800 2.53839900

1n:

PBE1PBE/6-31G(d): -2797.99971 a.u.

Point group: C_2

Cartesian coordinates:

C 0.00886700 1.35820200 0.97843300
C 0.00000000 0.75093300 -0.23976200

C 0.00000000 -0.75093300 -0.23976200
C -0.00886700 -1.35820200 0.97843300
Si 0.00000000 0.00000000 2.28180100
H -1.21946100 -0.00140100 3.12970200
H 1.21946100 0.00140100 3.12970200
Si -0.00926300 3.14698000 1.40168700
Si 0.01886900 1.75564900 -1.80443900
Si -0.01886900 -1.75564900 -1.80443900
Si 0.00926300 -3.14698000 1.40168700
F 0.94678200 1.03205900 -2.87246200
F -1.40943300 2.00453600 -2.43921600
F 0.66069700 3.16697100 -1.45480300
F 1.40312700 3.85535700 1.27483400
F -1.08785900 3.96012800 0.56700600
F -0.42892000 3.22082700 2.93621800
F 1.08785900 -3.96012800 0.56700600
F -1.40312700 -3.85535700 1.27483400
F 0.42892000 -3.22082700 2.93621800
F 1.40943300 -2.00453600 -2.43921600
F -0.94678200 -1.03205900 -2.87246200
F -0.66069700 -3.16697100 -1.45480300

1o:

PBE1PBE/6-31G(d): -1607.35242 a.u.

Point group: C_2

Cartesian coordinates:

C 0.00623100 1.37117100 0.71978000
C 0.00110300 0.75370600 -0.49711500
C -0.00110300 -0.75370600 -0.49711500
C -0.00623100 -1.37117100 0.71978000
Si 0.00000000 0.00000000 1.99733300
H -1.20702600 0.00693400 2.87427800
H 1.20702600 -0.00693400 2.87427800
Si 0.00000000 3.20130500 1.09881600
H -1.09909900 3.92034900 0.39361600
H -0.20459000 3.36899600 2.56533800
H 1.29352000 3.84803000 0.73646700
Si -0.00569100 1.73083800 -2.11519200
H 0.44058100 3.12218500 -1.83838100
H 0.92751700 1.11132700 -3.09801300
H -1.36584600 1.76853500 -2.72136600
Si 0.00569100 -1.73083800 -2.11519200
H 1.36584600 -1.76853500 -2.72136600
H -0.44058100 -3.12218500 -1.83838100
H -0.92751700 -1.11132700 -3.09801300
Si 0.00000000 -3.20130500 1.09881600
H -1.29352000 -3.84803000 0.73646700
H 1.09909900 -3.92034900 0.39361600
H 0.20459000 -3.36899600 2.56533800

1p:

PBE1PBE/6-31G(d): -666.32756 a.u.

Point group: C_1

Cartesian coordinates:

C -1.37248000 -0.40697700 -0.00726800
C -0.68915200 0.77834700 -0.00017700
C 0.79021100 0.73592600 -0.00537400
C 1.34224400 -0.50543900 0.01294600
Si -0.09106000 -1.73829100 0.01130800
H -0.00584600 -2.64479100 -1.17868000
H -0.08754900 -2.63363200 1.20977100
N 2.69908900 -0.72351700 0.06555900
H 3.03076800 -1.58358200 -0.34619100
H 3.23752600 0.09190500 -0.21437000
N 1.60541300 1.90256800 -0.05256500
H 1.19404200 2.62517600 -0.63577200
H 1.78625700 2.29769200 0.86702600
N -1.31738400 2.00150500 -0.05986900
H -2.31396400 1.90486200 0.11372500
H -0.87979300 2.73962400 0.47550800
N -2.80663100 -0.37753600 0.00466600
H -3.19245200 -0.70398300 -0.87852800
H -3.18249200 -0.98947400 0.72388800

2a:

PBE1PBE/6-31G(d): -735.62656 a.u.

Point group: D_{2h}

Cartesian coordinates:

C 0.67280800 1.53851300 0.00000000
C -0.67280800 1.53851300 0.00000000
C -0.67280800 -1.53851300 0.00000000
C 0.67280800 -1.53851300 0.00000000
H 1.18930500 2.50288400 0.00000000
H -1.18930500 2.50288400 0.00000000
H -1.18930500 -2.50288400 0.00000000
H 1.18930500 -2.50288400 0.00000000
Si 1.73805300 0.00000000 0.00000000
H 2.63612100 0.00000000 1.19403000
H 2.63612100 0.00000000 -1.19403000
Si -1.73805300 0.00000000 0.00000000
H -2.63612100 0.00000000 -1.19403000
H -2.63612100 0.00000000 1.19403000

2b:

PBE1PBE/6-31G(d): -1132.59367 a.u.

Point group: D_{2h}

Cartesian coordinates:

C -0.67284700 1.54804200 0.00000000
C 0.67284700 1.54804200 0.00000000
C 0.67284700 -1.54804200 0.00000000
C -0.67284700 -1.54804200 0.00000000
H -1.20135900 2.50444600 0.00000000
H 1.20135900 2.50444600 0.00000000
H 1.20135900 -2.50444600 0.00000000
H -1.20135900 -2.50444600 0.00000000
Si -1.69203100 0.00000000 0.00000000
Si 1.69203100 0.00000000 0.00000000
F -2.66401900 0.00000000 1.27635300
F -2.66401900 0.00000000 -1.27635300
F 2.66401900 0.00000000 1.27635300
F 2.66401900 0.00000000 -1.27635300

2c:

PBE1PBE/6-31G(d): -2573.68780 a.u.

Point group: D_{2h}

Cartesian coordinates:

C -0.67263700 0.00000000 1.54554700
C 0.67263700 0.00000000 1.54554700
C 0.67263700 0.00000000 -1.54554700
C -0.67263700 0.00000000 -1.54554700
H -1.20334800 0.00000000 2.50039100
H 1.20334800 0.00000000 2.50039100
H 1.20334800 0.00000000 -2.50039100
H -1.20334800 0.00000000 -2.50039100
Si -1.70281900 0.00000000 0.00000000
Si 1.70281900 0.00000000 0.00000000
Cl -2.92026200 1.66183900 0.00000000
Cl -2.92026200 -1.66183900 0.00000000
Cl 2.92026200 -1.66183900 0.00000000
Cl 2.92026200 1.66183900 0.00000000

2d:

PBE1PBE/6-31G(d): -892.82783 a.u.

Point group: D_{2h}

Cartesian coordinates:

C -0.67342600 0.00000000 1.52891300
C 0.67342600 0.00000000 1.52891300
C 0.67342600 0.00000000 -1.52891300
C -0.67342600 0.00000000 -1.52891300
H -1.18532200 0.00000000 2.49826500
H 1.18532200 0.00000000 2.49826500
H 1.18532200 0.00000000 -2.49826500
H -1.18532200 0.00000000 -2.49826500
Si -1.76094000 0.00000000 0.00000000
Si 1.76094000 0.00000000 0.00000000
C -2.86296200 1.53405900 0.00000000
H -3.51041300 1.55434600 -0.88453900
H -3.51041300 1.55434600 0.88453900

H -2.26402300 2.45086100 0.00000000
C -2.86296200 -1.53405900 0.00000000
H -3.51041300 -1.55434600 0.88453900
H -3.51041300 -1.55434600 -0.88453900
H -2.26402300 -2.45086100 0.00000000
C 2.86296200 -1.53405900 0.00000000
H 3.51041300 -1.55434600 -0.88453900
H 3.51041300 -1.55434600 0.88453900
H 2.26402300 -2.45086100 0.00000000
C 2.86296200 1.53405900 0.00000000
H 3.51041300 1.55434600 0.88453900
H 3.51041300 1.55434600 -0.88453900
H 2.26402300 2.45086100 0.00000000

2e:

PBE1PBE/6-31G(d): -2082.60658 a.u.

Point group: C_2

Cartesian coordinates:

C 0.00072000 0.67299700 -1.54836900
C -0.00072000 -0.67299700 -1.54836900
C 0.00071800 -0.67299700 1.54835700
C -0.00071800 0.67299700 1.54835700
H 0.00057000 1.19779200 -2.50661700
H -0.00057000 -1.19779200 -2.50661700
H 0.00056600 -1.19779500 2.50660300
H -0.00056600 1.19779500 2.50660300
Si 0.00000200 1.70419300 -0.00000600
Si -0.00000200 -1.70419300 -0.00000600
C -1.53554900 2.87341700 -0.01602000
C 1.53555100 2.87341900 0.01602200
C 1.53554900 -2.87341700 -0.01602000
C -1.53555100 -2.87341900 0.01602200
F -1.61940100 3.56755600 -1.16607200
F -1.49938600 3.76501300 0.99153500
F -2.67660400 2.17116200 0.11091400
F 1.49938600 3.76503000 -0.99152000
F 1.61940000 3.56754000 1.16608400
F 2.67660900 2.17117000 -0.11092200
F 2.67660400 -2.17116200 0.11091400
F 1.49938600 -3.76501300 0.99153500
F 1.61940100 -3.56755600 -1.16607200
F -1.49938600 -3.76503000 -0.99152000
F -1.61940000 -3.56754000 1.16608400
F -2.67660900 -2.17117000 -0.11092200

2f:

PBE1PBE/6-31G(d): -3088.55474 a.u.

Point group: D_2

Cartesian coordinates:

C -1.54556700 0.00302800 0.67258900
C -1.54556700 -0.00302800 -0.67258900
C 1.54556700 0.00302800 -0.67258900
C 1.54556700 -0.00302800 0.67258900
H -2.50686900 0.00314700 1.19224200
H -2.50686900 -0.00314700 -1.19224200
H 2.50686900 0.00314700 -1.19224200
H 2.50686900 -0.00314700 1.19224200
Si 0.00000000 0.00000000 1.72618800
Si 0.00000000 0.00000000 -1.72618800
F -1.51786300 -2.11096200 3.68323700
F 0.91502100 -1.63211600 4.38448900
F 0.43043000 -3.17717800 2.38453100
F -0.91502100 1.63211600 4.38448900
F 1.51786300 2.11096200 3.68323700
F -0.43043000 3.17717800 2.38453100
F 0.43043000 3.17717800 -2.38453100
F 0.91502100 1.63211600 -4.38448900
F -1.51786300 2.11096200 -3.68323700
F -0.91502100 -1.63211600 -4.38448900
F 1.51786300 -2.11096200 -3.68323700
F -0.43043000 -3.17717800 -2.38453100
Si -0.04569400 -1.85198300 3.13005000
Si 0.04569400 1.85198300 3.13005000
Si -0.04569400 1.85198300 -3.13005000

Si 0.04569400 -1.85198300 -3.13005000

2g:

PBE1PBE/6-31G(d): -1897.93995 a.u.

Point group: D_{2h}

Cartesian coordinates:

C -0.67345800 0.00000000 1.53504900
C 0.67345800 0.00000000 1.53504900
C 0.67345800 0.00000000 -1.53504900
C -0.67345800 0.00000000 -1.53504900
H -1.18835900 0.00000000 2.50043400
H 1.18835900 0.00000000 2.50043400
H 1.18835900 0.00000000 -2.50043400
H -1.18835900 0.00000000 -2.50043400
Si -1.75264800 0.00000000 0.00000000
Si 1.75264800 0.00000000 0.00000000
Si -3.13189300 1.90299100 0.00000000
Si -3.13189300 -1.90299100 0.00000000
Si 3.13189300 -1.90299100 0.00000000
Si 3.13189300 1.90299100 0.00000000
H -4.00968000 1.91265400 1.20728100
H -4.00968000 1.91265400 -1.20728100
H -2.32158100 3.15527300 0.00000000
H 2.32158100 3.15527300 0.00000000
H 4.00968000 1.91265400 -1.20728100
H 4.00968000 1.91265400 1.20728100
H 4.00968000 -1.91265400 1.20728100
H 2.32158100 -3.15527300 0.00000000
H 4.00968000 -1.91265400 -1.20728100
H -2.32158100 -3.15527300 0.00000000
H -4.00968000 -1.91265400 -1.20728100
H -4.00968000 -1.91265400 1.20728100

2h:

PBE1PBE/6-31G(d): -2369.27173 a.u.

Point group: D_2

Cartesian coordinates:

C -1.52780000 0.00196000 0.67426500
C -1.52780000 -0.00196000 -0.67426500
C 1.52780000 0.00196000 -0.67426500
C 1.52780000 -0.00196000 0.67426500
H -2.49747400 0.00411000 1.18437600
H -2.49747400 -0.00411000 -1.18437600
H 2.49747400 0.00411000 -1.18437600
H 2.49747400 -0.00411000 1.18437600
Si 0.00000000 0.00000000 1.77343600
Si 0.00000000 0.00000000 -1.77343600
Si 0.01352700 -1.94807500 3.11545000
Si -0.01352700 1.94807500 3.11545000
Si 0.01352700 1.94807500 -3.11545000
Si -0.01352700 -1.94807500 -3.11545000
C 0.43629700 3.45387600 2.06024000
H 1.42689900 3.33714400 1.60632200
H -0.28525400 3.59605200 1.24798300
H 0.44716400 4.36873000 2.66569400
C 1.23889500 1.79900100 4.53023800
H 1.25159000 2.71690800 5.13108800
H 0.99419600 0.96714400 5.20077300
H 2.25525200 1.63618800 4.15356900
C -1.74132600 2.20883600 3.84885400
H -2.05022600 1.36423700 4.47469500
H -1.76345700 3.11182100 4.47155300
H -2.49113200 2.33164800 3.05902700
C 1.74132600 -2.20883600 3.84885400
H 2.05022600 -1.36423700 4.47469500
H 1.76345700 -3.11182100 4.47155300
H 2.49113200 -2.33164800 3.05902700
C -1.23889500 -1.79900100 4.53023800
H -1.25159000 -2.71690800 5.13108800
H -0.99419600 -0.96714400 5.20077300
H -2.25525200 -1.63618800 4.15356900
C -0.43629700 -3.45387600 2.06024000
H -1.42689900 -3.33714400 1.60632200
H 0.28525400 -3.59605200 1.24798300

H -0.44716400 -4.36873000 2.66569400
C -0.43629700 3.45387600 -2.06024000
H -1.42689900 3.33714400 -1.60632200
H 0.28525400 3.59605200 -1.24798300
H -0.44716400 4.36873000 -2.66569400
C 1.74132600 2.20883600 -3.84885400
H 2.05022600 1.36423700 -4.47469500
H 1.76345700 3.11182100 -4.47155300
H 2.49113200 2.33164800 -3.05902700
C -1.23889500 1.79900100 -4.53023800
H -1.25159000 2.71690800 -5.13108800
H -0.99419600 0.96714400 -5.20077300
H -2.25525200 1.63618800 -4.15356900
C 1.23889500 -1.79900100 -4.53023800
H 1.25159000 -2.71690800 -5.13108800
H 0.99419600 -0.96714400 -5.20077300
H 2.25525200 -1.63618800 -4.15356900
C -1.74132600 -2.20883600 -3.84885400
H -2.05022600 -1.36423700 -4.47469500
H -1.76345700 -3.11182100 -4.47155300
H -2.49113200 -2.33164800 -3.05902700
C 0.43629700 -3.45387600 -2.06024000
H 1.42689900 -3.33714400 -1.60632200
H -0.28525400 -3.59605200 -1.24798300
H 0.44716400 -4.36873000 -2.66569400

2i:

PBE1PBE/6-31G(d): -957.01293 a.u.

Point group: D_2

Cartesian coordinates:

C -0.01062000 1.52545600 -0.67365700
C 0.01062000 1.52545600 0.67365700
C -0.01062000 -1.52545600 0.67365700
C 0.01062000 -1.52545600 -0.67365700
H -0.02007900 2.49245300 -1.19011300
H 0.02007900 2.49245300 1.19011300
H -0.02007900 -2.49245300 1.19011300
H 0.02007900 -2.49245300 -1.19011300
Si 0.00000000 0.00000000 -1.76274400
Si 0.00000000 0.00000000 1.76274400
N -1.34701800 0.20820400 -2.83378600
H -2.21681600 0.55915900 -2.45518600
H -1.51851900 -0.50378400 -3.53233400
N 1.34701800 -0.20820400 -2.83378600
H 2.21681600 -0.55915900 -2.45518600
H 1.51851900 0.50378400 -3.53233400
N 1.34701800 0.20820400 2.83378600
H 1.51851900 -0.50378400 3.53233400
H 2.21681600 0.55915900 2.45518600
N -1.34701800 -0.20820400 2.83378600
H -2.21681600 -0.55915900 2.45518600
H -1.51851900 0.50378400 3.53233400

2j:

PBE1PBE/6-31G(d): -1132.27222 a.u.

Point group: D_{2h}

Cartesian coordinates:

C -1.51781600 0.66967000 0.00000000
C -1.51781600 -0.66967000 0.00000000
C 1.51781600 -0.66967000 0.00000000
C 1.51781600 0.66967000 0.00000000
Si 0.00000000 1.77376600 0.00000000
Si 0.00000000 -1.77376600 0.00000000
H 0.00000000 -2.63789500 1.20799500
H 0.00000000 -2.63789500 -1.20799500
H 0.00000000 2.63789500 -1.20799500
H 0.00000000 2.63789500 1.20799500
F 2.69342600 -1.33152500 0.00000000
F 2.69342600 1.33152500 0.00000000
F -2.69342600 1.33152500 0.00000000
F -2.69342600 -1.33152500 0.00000000

2k:

PBE1PBE/6-31G(d): -2573.46601 a.u.

Point group: D_{2h}

Cartesian coordinates:

C -1.54105000 0.67066200 0.00000000
C -1.54105000 -0.67066200 0.00000000
C 1.54105000 -0.67066200 0.00000000
C 1.54105000 0.67066200 0.00000000
Si 0.00000000 1.74630200 0.00000000
Si 0.00000000 -1.74630200 0.00000000
H 0.00000000 -2.60030300 1.21353200
H 0.00000000 -2.60030300 -1.21353200
H 0.00000000 2.60030300 -1.21353200
H 0.00000000 2.60030300 1.21353200
Cl 3.00748100 -1.60651300 0.00000000
Cl 3.00748100 1.60651300 0.00000000
Cl -3.00748100 1.60651300 0.00000000
Cl -3.00748100 -1.60651300 0.00000000

2l:

PBE1PBE/6-31G(d): -892.78180 a.u.

Point group: D_{2h}

Cartesian coordinates:

C -1.57418500 0.67629900 0.00000000
C -1.57418500 -0.67629900 0.00000000
C 1.57418500 -0.67629900 0.00000000
C 1.57418500 0.67629900 0.00000000
Si 0.00000000 1.68842200 0.00000000
Si 0.00000000 -1.68842200 0.00000000
H 0.00000000 -2.60154000 1.18705500
H 0.00000000 -2.60154000 -1.18705500
H 0.00000000 2.60154000 -1.18705500
H 0.00000000 2.60154000 1.18705500
C 2.86803700 -1.45934900 0.00000000
H 3.47705800 -1.21990900 -0.88179900
H 3.47705800 -1.21990900 0.88179900
H 2.70567700 -2.54243400 0.00000000
C 2.86803700 1.45934900 0.00000000
H 3.47705800 1.21990900 0.88179900
H 3.47705800 1.21990900 -0.88179900
H 2.70567700 2.54243400 0.00000000
C -2.86803700 1.45934900 0.00000000
H -3.47705800 1.21990900 -0.88179900
H -3.47705800 1.21990900 0.88179900
H -2.70567700 2.54243400 0.00000000
C -2.86803700 -1.45934900 0.00000000
H -3.47705800 -1.21990900 0.88179900
H -3.47705800 -1.21990900 -0.88179900
H -2.70567700 -2.54243400 0.00000000

2m:

PBE1PBE/6-31G(d): -2082.60716 a.u.

Point group: C_2

Cartesian coordinates:

C -0.01714500 0.67189600 -1.55494700
C 0.01714500 -0.67189600 -1.55494700
C 0.01712800 -0.67189400 1.55492700
C -0.01712800 0.67189400 1.55492700
Si 0.01712800 1.74924500 -0.00000800
Si -0.01712800 -1.74924500 -0.00000800
H -1.27987400 -2.52389000 -0.00003000
H 1.15827700 -2.65287200 0.00001200
H 1.27987400 2.52389000 -0.00003000
H -1.15827700 2.65287200 0.00001200
C 0.08024900 -1.50469100 2.82335000
C -0.08024900 1.50469100 2.82335000
C -0.08022400 1.50472600 -2.82335300
C 0.08022400 -1.50472600 -2.82335300
F -0.24873600 -2.78294700 2.53139100
F 1.31826700 -1.52918500 3.32690200
F -0.75799400 -1.09900800 3.77824600
F -1.31826700 1.52918500 3.32690200
F 0.75799400 1.09900800 3.77824600
F 0.24873600 2.78294700 2.53139100
F 0.24841700 2.78304100 -2.53127300
F -1.31816100 1.52897000 -3.32711800

F 0.75829300 1.09930300 -3.77811900
F -0.75829300 -1.09930300 -3.77811900
F 1.31816100 -1.52897000 -3.32711800
F -0.24841700 -2.78304100 -2.53127300

2n:

PBE1PBE/6-31G(d): -3088.55133 a.u.

Point group: D_2

Cartesian coordinates:

C 1.57179000 0.02077900 0.67974700
C 1.57179000 -0.02077900 -0.67974700
C -1.57179000 0.02077900 -0.67974700
C -1.57179000 -0.02077900 0.67974700
Si 0.00000000 0.00000000 1.73117100
Si 0.00000000 0.00000000 -1.73117100
H -0.00186000 1.20712300 -2.59763800
H 0.00186000 -1.20712300 -2.59763800
H -0.00186000 -1.20712300 2.59763800
H 0.00186000 1.20712300 2.59763800
Si -3.12876700 -0.07079700 1.69704200
Si -3.12876700 0.07079700 -1.69704200
Si 3.12876700 -0.07079700 -1.69704200
Si 3.12876700 0.07079700 1.69704200
F -2.76656900 0.47387100 3.14789800
F -3.72418000 -1.52902600 1.88059600
F -4.24649000 0.87414800 1.07596700
F -4.24649000 -0.87414800 -1.07596700
F -3.72418000 1.52902600 -1.88059600
F -2.76656900 -0.47387100 -3.14789800
F 2.76656900 0.47387100 -3.14789800
F 3.72418000 -1.52902600 -1.88059600
F 4.24649000 0.87414800 -1.07596700
F 4.24649000 -0.87414800 1.07596700
F 3.72418000 1.52902600 1.88059600
F 2.76656900 -0.47387100 3.14789800

2o:

PBE1PBE/6-31G(d): -1897.90629 a.u.

Point group: D_2

Cartesian coordinates:

C 1.58089200 0.00687100 0.68027300
C 1.58089200 -0.00687100 -0.68027300
C -1.58089200 0.00687100 -0.68027300
C -1.58089200 -0.00687100 0.68027300
Si 0.00000000 0.00000000 1.70152300
Si 0.00000000 0.00000000 -1.70152300
H -0.00180900 1.19493400 -2.59784800
H 0.00180900 -1.19493400 -2.59784800
H -0.00180900 -1.19493400 2.59784800
H 0.00180900 1.19493400 2.59784800
Si -3.16818900 -0.03601700 1.71215400
Si -3.16818900 0.03601700 -1.71215400
Si 3.16818900 -0.03601700 -1.71215400
Si 3.16818900 0.03601700 1.71215400
H -2.84886300 0.40272500 3.09971200
H -3.73602500 -1.41253400 1.77526500
H -4.19500700 0.88286800 1.14394000
H -4.19500700 -0.88286800 -1.14394000
H -3.73602500 1.41253400 -1.77526500
H -2.84886300 -0.40272500 -3.09971200
H 2.84886300 0.40272500 -3.09971200
H 3.73602500 -1.41253400 -1.77526500
H 4.19500700 -0.88286800 1.14394000
H 2.84886300 -0.40272500 3.09971200
H 3.73602500 1.41253400 1.77526500
H 4.19500700 0.88286800 -1.14394000

2p:

PBE1PBE/6-31G(d): -956.88898 a.u.

Point group: C_s

Cartesian coordinates:

C 0.68363800 -0.03399200 1.54979300
C -0.67587700 -0.04245400 1.54598200
C -0.67587700 -0.04245400 -1.54598200

C 0.68363800 -0.03399200 -1.54979300
Si 1.69496400 0.24253600 0.00000000
Si -1.68748400 0.17318400 0.00000000
N -1.32829500 -0.19770200 2.80572500
H -1.18190800 0.60770900 3.41030100
H -2.32532600 -0.35759300 2.72198700
N 1.37420000 -0.13196000 -2.76071300
H 2.29422600 -0.54832400 -2.68850400
H 0.81150500 -0.60184800 -3.46768600
N 1.37420000 -0.13196000 2.76071300
H 0.81150500 -0.60184800 3.46768600
H 2.29422600 -0.54832400 2.68850400
N -1.32829500 -0.19770200 -2.80572500
H -2.32532600 -0.35759300 -2.72198700
H -1.18190800 0.60770900 -3.41030100
H -2.79535900 -0.84027300 0.00000000
H -2.40644000 1.49063900 0.00000000
H 2.32902500 1.59477200 0.00000000
H 2.83525100 -0.73249600 0.00000000

3a:
PBE1PBE/6-31G(d): -887.88012 a.u.
Point group: D_{2h}
Cartesian coordinates:
C -1.91875300 1.40178600 0.00000000
C -0.74518400 0.74806700 0.00000000
C -0.74518400 -0.74806700 0.00000000
C -1.91875300 -1.40178600 0.00000000
H -2.04610500 2.47940000 0.00000000
H -2.04610500 -2.47940000 0.00000000
Si -3.18535900 0.00000000 0.00000000
C 0.74518400 0.74806700 0.00000000
C 0.74518400 -0.74806700 0.00000000
C 1.91875300 1.40178600 0.00000000
H 2.04610500 2.47940000 0.00000000
C 1.91875300 -1.40178600 0.00000000
H 2.04610500 -2.47940000 0.00000000
Si 3.18535900 0.00000000 0.00000000
H 4.07041200 0.00000000 1.20279800
H 4.07041200 0.00000000 -1.20279800
H -4.07041200 0.00000000 -1.20279800
H -4.07041200 0.00000000 1.20279800

3b:
PBE1PBE/6-31G(d): -1284.75765 a.u.
Point group: D_{2h}
Cartesian coordinates:
C 1.90752500 1.41674600 0.00000000
C 0.74244400 0.75580200 0.00000000
C 0.74244400 -0.75580200 0.00000000
C 1.90752500 -1.41674600 0.00000000
H 2.04051400 2.49269500 0.00000000
H 2.04051400 -2.49269500 0.00000000
Si 3.13868900 0.00000000 0.00000000
C -0.74244400 0.75580200 0.00000000
C -0.74244400 -0.75580200 0.00000000
C -1.90752500 1.41674600 0.00000000
H -2.04051400 2.49269500 0.00000000
C -1.90752500 -1.41674600 0.00000000
H -2.04051400 -2.49269500 0.00000000
Si -3.13868900 0.00000000 0.00000000
F 4.10664800 0.00000000 1.27567700
F 4.10664800 0.00000000 -1.27567700
F -4.10664800 0.00000000 1.27567700
F -4.10664800 0.00000000 -1.27567700

3h:
PBE1PBE/6-31G(d): -2521.45264 a.u.
Point group: C_1
Cartesian coordinates:
C 1.93073900 -0.11295900 -1.38749000
C 0.74750100 -0.13508200 -0.74034100
C 0.74466300 -0.13471400 0.74344900
C 1.92515700 -0.11094800 1.39563400

H 2.05755400 -0.10084700 -2.46620900
H 2.04733100 -0.10009400 2.47489900
Si 3.21326200 -0.06193500 0.00685100
C -0.74466500 -0.13473200 -0.74344300
C -0.74750400 -0.13506300 0.74034600
C -1.92515900 -0.11098100 -1.39562900
H -2.04733300 -0.10015600 -2.47489400
C -1.93074200 -0.11292300 1.38749500
H -2.05755700 -0.10078300 2.46621400
Si -3.21326500 -0.06193300 -0.00684800
Si -4.61754600 -1.96074400 -0.03294000
Si 4.32679700 2.02126500 -0.03211700
Si 4.61755100 -1.96074000 0.03296200
Si -4.32680200 2.02126600 0.03209300
C -3.55551500 -3.51028200 0.17886400
H -4.17464200 -4.41574600 0.15937100
H -2.81338400 -3.58963400 -0.62293200
H -3.01277800 -3.48987100 1.12997100
C -5.53791400 -2.05210300 -1.68530700
H -4.83853800 -2.11539600 -2.52636600
H -6.17962700 -2.94140600 -1.71784100
H -6.17455800 -1.17474300 -1.84518700
C -3.05202700 3.38729100 -0.25347700
H -2.58878000 3.29490400 -1.24154600
H -3.51828600 4.37804300 -0.18647900
H -2.25081600 3.33745300 0.49189000
C -5.65403000 2.10830900 -1.31727700
H -6.12015500 3.10150400 -1.33073900
H -5.23074300 1.92662200 -2.31151700
H -6.44873400 1.37186300 -1.15160600
C 3.05202100 3.38729300 0.25343300
H 2.58876400 3.29491000 1.24149900
H 3.51828100 4.37804400 0.18643600
H 2.25081600 3.33745200 -0.49194000
C 5.65401800 2.10831800 1.31726000
H 6.12013100 3.10151900 1.33073200
H 5.23072800 1.92662000 2.31149700
H 6.44873100 1.37188300 1.15158800
C 5.53793200 -2.05208700 1.68532200
H 4.83856400 -2.11538300 2.52638700
H 6.17965200 -2.94138600 1.71785300
H 6.17457200 -1.17472300 1.84519300
C 3.55552700 -3.51028500 -0.17882600
H 4.17466100 -4.41574500 -0.15934600
H 2.81341100 -3.58964500 0.62298300
H 3.01277300 -3.48987700 -1.12992400
C -5.14679300 2.27314200 1.72033500
H -5.88619200 1.49372300 1.93532900
H -4.40441600 2.26101300 2.52602300
H -5.66196700 3.24120700 1.75574500
C -5.88157400 -1.86438600 1.37413300
H -5.39206000 -1.78097600 2.35086100
H -6.54719200 -1.00145600 1.25788900
H -6.50637500 -2.76611900 1.39111500
C 5.88156700 -1.86438000 -1.37412100
H 5.39204600 -1.78094600 -2.35084400
H 6.54719700 -1.00146000 -1.25786900
H 6.50635700 -2.76612000 -1.39112300
C 5.14679900 2.27313000 -1.72035600
H 5.88619400 1.49370500 -1.93534300
H 4.40442700 2.26100500 -2.52604800
H 5.66198100 3.24119100 -1.75576400

Silole with SiF($SiMe_3$) moiety:
PBE1PBE/6-31G(d): -952.75657 a.u.
Point group: C_s
Cartesian coordinates:
C -1.12415100 1.34325000 1.35411300
C -1.12415100 2.54422200 0.74156800
C -1.12415100 2.54422200 -0.74156800
C -1.12415100 1.34325000 -1.35411300
H -1.10843400 1.24128100 2.43454100
H -1.12017100 3.49608100 1.27285600
H -1.12017100 3.49608100 -1.27285600

H -1.10843400 1.24128100 -2.43454100
Si -0.94985200 0.06298700 0.00000000
Si 1.16003000 -0.97076700 0.00000000
C 1.34731800 -2.04514000 1.54523700
H 0.57475800 -2.82058100 1.59007700
H 2.32372800 -2.54497400 1.54851600
H 1.27650800 -1.44713500 2.46054500
C 1.34731800 -2.04514000 -1.54523700
H 2.32372800 -2.54497400 -1.54851600
H 0.57475800 -2.82058100 -1.59007700
H 1.27650800 -1.44713500 -2.46054500
C 2.47266800 0.38904400 0.00000000
H 2.38081200 1.02901600 0.88418700
H 3.48034300 -0.04383000 0.00000000
H 2.38081200 1.02901600 -0.88418700
F -2.12238100 -1.06632100 0.00000000

1,4-Disilacyclohexa-2,5-diene with SiF($SiMe_3$) moiety (Z-isomer):
PBE1PBE/6-31G(d): -1750.92456 a.u.
Point group: C_2
Cartesian coordinates:
C -1.30185800 1.04765600 -1.17695300
C -1.64916800 -0.25390000 -1.17139300
C 1.30185800 -1.04765600 -1.17695300
C 1.64916800 0.25390000 -1.17139300
H -2.09618300 1.79683900 -1.25710900
H -2.71133800 -0.50834400 -1.24683600
H 2.09618300 -1.79683900 -1.25710900
H 2.71133800 0.50834400 -1.24683600
Si 0.45119000 1.68464800 -1.03528700
Si -0.45119000 -1.68464800 -1.03528700
Si 0.78647100 2.97221400 0.90219200
Si -0.78647100 -2.97221400 0.90219200
C 2.62224000 3.40138900 1.06190000
H 2.98048200 3.95706100 0.18824000
H 2.79515700 4.02490700 1.94760000
H 3.23880600 2.50133000 1.16451400
C -0.22687500 4.56376100 0.75979800
H -0.04768000 5.21395500 1.62484200
H 0.03966500 5.12417600 -0.14287900
H -1.30196900 4.35500500 0.71927300
C 0.22687500 2.00300500 2.42592900
H -0.83184200 1.72861600 2.35661000
H 0.80393000 1.08056900 2.55321000
H 0.35677000 2.60576400 3.33295400
C 0.22687500 -4.56376100 0.75979800
H 0.04768000 -5.21395500 1.62484200
H -0.03966500 -5.12417600 -0.14287900
H 1.30196900 -4.35500500 0.71927300
C -2.62224000 -3.40138900 1.06190000
H -2.98048200 -3.95706100 0.18824000
H -2.79515700 -4.02490700 1.94760000
H -3.23880600 -2.50133000 1.16451400
C -0.22687500 -2.00300500 2.42592900
H 0.83184200 -1.72861600 2.35661000
H -0.80393000 -1.08056900 2.55321000
H -0.35677000 -2.60576400 3.33295400
F -0.72569600 -2.67302900 -2.30808700
F 0.72569600 2.67302900 -2.30808700

1,4-Disilacyclohexa-2,5-diene with SiF($SiMe_3$) moiety (E-isomer):
PBE1PBE/6-31G(d): -1750.92508 a.u.
Point group: C_2
Cartesian coordinates:
C 0.33021700 0.58765100 -1.53016400
C -0.33021700 -0.58765100 -1.53016400
C -0.33016500 -0.58767700 1.53103700
C 0.33016500 0.58767700 1.53103700
H 0.58171000 1.03984400 -2.49540000
H -0.58171000 -1.03984400 -2.49540000
H -0.58161800 -1.03990300 2.49626600
H 0.58161800 1.03990300 2.49626600

Si 0.76206900 1.57044300 0.00043700
Si -0.76206900 -1.57044300 0.00043700
Si -0.33016500 3.65006900 -0.00016900
Si 0.33016500 -3.65006900 -0.00016900
C 0.14848100 4.62842600 1.54701500
H 1.22928000 4.80083200 1.59475300
H -0.34732100 5.60685400 1.55046000
H -0.14944300 4.10376500 2.46185500
C 0.14959600 4.62745500 -1.54764400
H -0.34616500 5.60590100 -1.55203400
H 1.23043100 4.79978900 -1.59480300
H -0.14776800 4.10221100 -2.46233200
C -2.19349200 3.33018200 -0.00093700
H -2.50066100 2.75985500 -0.88466200
H -2.50174900 2.76096600 0.88311900
H -2.74984300 4.27541800 -0.00188900
C 2.19349200 -3.33018200 -0.00093700
H 2.50066100 -2.75985500 -0.88466200
H 2.50174900 -2.76096600 0.88311900
H 2.74984300 -4.27541800 -0.00188900
C -0.14848100 -4.62842600 1.54701500
H -1.22928000 -4.80083200 1.59475300
H 0.34732100 -5.60685400 1.55046000
H 0.14944300 -4.10376500 2.46185500
C -0.14959600 -4.62745500 -1.54764400
H 0.34616500 -5.60590100 -1.55203400
H -1.23043100 -4.79978900 -1.59480300
H 0.14776800 -4.10221100 -2.46233200
F -2.37702700 -1.82553600 0.00056400
F 2.37702700 1.82553600 0.00056400

1,4-Disilacyclohexa-2,5-diene with one SiF_2 and one $Si(SiMe_3)_2$ moiety:
PBE1PBE/6-31G(d): -1750.93752 a.u.
Point group: C_2
Cartesian coordinates:
C 1.28320600 -0.83415900 0.75581100
C 1.29277900 -0.83662500 2.10466700
C -1.29277900 0.83662500 2.10466700
C -1.28320600 0.83415900 0.75581100
H 2.09383600 -1.35932800 0.23954700
H 2.10061700 -1.35825600 2.62572900
H -2.10061700 1.35825600 2.62572900
H -2.09383600 1.35932800 0.23954700
Si 0.00000000 0.00000000 -0.34229500
Si 0.00000000 0.00000000 3.12004500
Si -1.11276900 -1.64174200 -1.62681400
Si 1.11276900 1.64174200 -1.62681400
C 1.57220100 3.08587200 -0.49759700
H 0.67968600 3.54705700 -0.06086900
H 2.20985100 2.75470300 0.32967200
H 2.11594800 3.85966000 -1.05328200
C 0.00000000 2.25842400 -3.02907100
H 0.50769200 3.04348100 -3.60315900
H -0.25266100 1.45064200 -3.72562100
H -0.93773800 2.68026100 -2.65006500
C 2.68360700 0.89020000 -2.36903100
H 2.45950200 0.03754300 -3.01992500
H 3.21615300 1.63735500 -2.97044200
H 3.36893700 0.54503800 -1.58678800
C -2.68360700 -0.89020000 -2.36903100
H -2.45950200 -0.03754300 -3.01992500
H -3.21615300 -1.63735500 -2.97044200
H -3.36893700 -0.54503800 -1.58678800
C 0.00000000 -2.25842400 -3.02907100
H -0.50769200 -3.04348100 -3.60315900
H 0.25266100 -1.45064200 -3.72562100
H 0.93773800 -2.68026100 -2.65006500
C -1.57220100 -3.08587200 -0.49759700
H -0.67968600 -3.54705700 -0.06086900
H -2.20985100 -2.75470300 0.32967200
H -2.11594800 -3.85966000 -1.05328200
F -0.68847900 -1.06491400 4.11307400
F 0.68847900 1.06491400 4.11307400
